# Genome-Wide Identification and Expression Profiling Analysis of *ZmPIN*, *ZmPILS*, *ZmLAX* and *ZmABCB* Auxin Transporter Gene Families in Maize (*Zea mays* L.) under Various Abiotic Stresses

**DOI:** 10.1371/journal.pone.0118751

**Published:** 2015-03-05

**Authors:** Runqing Yue, Shuanggui Tie, Tao Sun, Lei Zhang, Yanjun Yang, Jianshuang Qi, Shufeng Yan, Xiaohua Han, Huizhong Wang, Chenjia Shen

**Affiliations:** 1 Henan Academy of Agricultural Sciences, Zhengzhou 450002, China; 2 College of Life and Environmental Sciences, Hangzhou Normal University, Hangzhou 310036, China; 3 Department of Plant Pathology, Washington State University, Pullman, WA 99164-6430, United States of America; Henan Agricultural Univerisity, CHINA

## Abstract

The auxin influx carriers auxin resistant 1/like aux 1 (AUX/LAX), efflux carriers pin-formed (PIN) (together with PIN-like proteins) and efflux/conditional P-glycoprotein (ABCB) are major protein families involved in auxin polar transport. However, how they function in responses to exogenous auxin and abiotic stresses in maize is largely unknown. In this work, the latest updated maize (*Zea mays* L.) reference genome sequence was used to characterize and analyze the *ZmLAX*, *ZmPIN*, *ZmPILS* and *ZmABCB* family genes from maize. The results showed that five *ZmLAXs*, fifteen *ZmPINs*, nine *ZmPILSs* and thirty-five *ZmABCBs* were mapped on all ten maize chromosomes. Highly diversified gene structures, nonconservative transmembrane helices and tissue-specific expression patterns suggested the possibility of function diversification for these genes. Quantitative real-time polymerase chain reaction (qRT-PCR) was used to analyze the expression patterns of *ZmLAX*, *ZmPIN*, *ZmPILS* and *ZmABCB* genes under exogenous auxin and different environmental stresses. The expression levels of most *ZmPIN*, *ZmPILS*, *ZmLAX* and *ZmABCB* genes were induced in shoots and were reduced in roots by various abiotic stresses (drought, salt and cold stresses). The opposite expression response patterns indicated the dynamic auxin transport between shoots and roots under abiotic stresses. Analysis of the expression patterns of *ZmPIN*, *ZmPILS*, *ZmLAX* and *ZmABCB* genes under drought, salt and cold treatment may help us to understand the possible roles of maize auxin transporter genes in responses and tolerance to environmental stresses.

## Introduction

Plants are constantly challenged by environmental stresses, such as high salinity, drought and cold. Many adaptive mechanisms at different levels, including molecular, cellular and physiological processes, have developed to help plants survive in adverse environmental conditions [1,2]. Auxin plays a critical role in the temporal coordination of plant tolerance to stress [3–5] and many environmental stress responses rely on the dynamic distribution of auxin within different plant tissues [6]. From its primary synthesis sites, such as apical meristems and developing leaf tips, auxin flows in an irreversible direction down towards the roots through the stem vascular tissues, and auxin transporters are thought to be involved in this process [7,8]. Auxin transport proteins in plants are grouped into three major families: auxin resistant 1/like aux1 (AUX1⁄LAX) influx carriers, pin-formed (PIN) (together with PIN-like) efflux carriers and P-glycoprotein (MDR⁄PGP⁄ABCB) efflux/conditional transporters [9,10].

The auxin influx carrier, AUX1, belongs to the amino acid permease family of proton-driven transporters, and functions in uptake of indole-3-acetic acid (IAA) into cells [11,12]. *LAX* genes, the paralogs of *AUX1*, maintain auxin distribution pattern against environmental or developmental influences [13]. In *Arabidopsis*, *AtLAX3* is reported to promote the initiation of lateral root primordia by increasing a selection of cell-wall-remodeling enzymes [14], and a wild cherry *Prunus avium* gene, *PaLAX1*, accelerates the uptake rate of auxin into cells and changes the distribution of free endogenous auxin [15].

The *PIN* gene family was first cloned in *Arabidopsis* and functioned as auxin efflux transporter encoding genes [16]. In dicotyledonous *Arabidopsis*, a number of *PIN* genes have been studied in detail. The *PIN* family genes are involved in various developmental processes, including phototropism (*AtPIN1*, *AtPIN3* and *AtPIN7*), vascular bundle differentiation (*AtPIN1*), apical embryonic structure specification (*AtPIN7*), alkaline-stress responses (*AtPIN2*), lateral root formation (*AtPIN2*), root hair growth (*AtPIN5*) and root pattern establishment (*AtPIN4*) [17–20]. The *PIN* family gene expression patterns are currently an important area of research. Previous studies have shown that *AtPIN1* and *AtPIN4* expression levels are upregulated by a MADS-domain transcription factor [21] and phytohormones, such as brassinosteroid, selectively down-regulate *AtPIN4* and *AtPIN7* expression [22]. Recently, the *ERECTA* family genes were found to be essential for *AtPIN1* expression during mid-vein formation of future leaf primordia [23]. The expression patterns of *ZmPIN1a* and *ZmPIN1b*, two novel putative orthologs of *AtPIN1* in maize, have also been analyzed during maize development. ZmPIN5b protein is localized on basal cell membranes and may be involved in vascular tissues differentiation [24]. PIN-LIKE proteins, which have a low (10%-18%) sequence identity with PINs, are the most recently characterized auxin transport family proteins in *Arabidopsis* and their involvement in auxin transport across the plasma membrane has been well studied in heterologous systems [10,25].

An ATP-binding cassette (ABC) transporter family has been reported to be responsible for auxin polar movement at the cell level [26]. In *Arabidopsis*, AtABCB1 and AtABCB19 are involved in auxin export and AtABCB4 catalyzes auxin import [27]. AtABCB1-mediated auxin efflux is modulated by two partners: PINOID (PID) and TWISTED DWARF1. The phosphorylation of PID determines the auxin transporter activity and enhances AtABCB1-mediated auxin efflux [28]. Furthermore, AtABCB19 participates in auxin-mediated differential tropic responses by interacting with PIN proteins [29]. An *Arabidopsis* plasma membrane-localized ABC transporter, ABCB21, has been identified as a facultative auxin importer/exporter and it is regulated by cytoplasmic auxin concentration [30]. A monocot rice gene, *OsABCB14*, has been reported to have a role in auxin transport and iron homeostasis, and this was the first evidence that an ABCB gene had been shown to be involved in iron uptake [31].

Recently, some auxin transporter family genes have been studied in other species, such as rice, *Sorghum bicolor*, *Populus trichocarpa* and *Prunus avium* [15,32–34]. Some *PIN* and *ABCB* family genes have already been identified in maize [24,35], but the underlying mechanism linking the auxin transporter family gene expression levels and abiotic stresses (salt, drought and cold stresses) in maize is largely unknown. Maize (*Zea mays* L.) is an important cereal crop, and is the staple food for many people, worldwide. Under natural conditions, high salinity, drought and cold are the major environmental stresses experienced by maize plants [36,37]. Our work provides comprehensive information on the *ZmPIN*, *ZmPILS*, *ZmLAX*, *ZmABCB* gene families and investigated the different spatio-temporal expression patterns of these genes under salt, drought and cold stresses. The distinctive spatio-temporal expression patterns of the *ZmPIN*, *ZmPILS*, *ZmLAX* and Zm*ABCB* genes, and their differential responses to salt, drought and cold stresses will become important research areas to increase abiotic stress tolerance in maize.

## Material and Methods

### Plant Material and stress treatments

Maize (*Zea mays* L. inbred line B73) seeds (wild-type) were used in this study. B73 seeds were surface sterilized, washed with sterile water, and germinated in petri plates in chamber overnight at 28°C with a photoperiod of 16-h light and 8-h dark and a relative humidity of 60%. Then the seedlings were transferred to nutrient solution (half-strong modified Hoagland), the pH of the nutrient solution was adjusted to 5.6, and nutrient solution was changed every 3 days. 14-day-old seedlings were used for RNA isolation and stress treatment experiments. Then shoots and roots samples of maize seedlings were collected for RNA isolation respectively. For auxin treatment on maize seedlings, 14-day-old seedlings were soaked in nutrient solution with or without (mock treatment) 10μM IAA for 48 hours, then roots and shoots of maize seedlings at different time points were collected for RNA isolation respectively. Experiment was repeated for 5 times with similar results. Stress treatments were performed as follows: in salt stress experiment, the roots of maize seedlings were immersed in nutrient solution containing 150 mM NaCl for 48 hours [37]; in drought tress experiment, maize seedlings were planted in sand with MS liquid medium for one week, and then not irrigated for 3 days as drought treatment [33]; for cold treatment, seedlings were put into a 4°C growth chamber for 48 hours [38]. Untreated seedlings were used as controls. Student *t*-test analysis between mock-inoculated plants and stress-inoculated plants was performed to reveal the differential expression patterns of *ZmPIN*, *ZmPILS*, *ZmLAX* and *ZmABCB* family genes.

### Identification of *PIN*, *PILS*, *AUX/LAX* and *ABCB* auxin transporter gene families in maize

The Hidden Markov Model (HMM) profiles of the *PIN*, *PILS*, *AUX/LAX* and *ABCB* auxin transporter gene families (Pfam 01490: Transmembrane amino acid transporter protein; Pfam PF03547: Membrane transport protein; Pfam 03547: Membrane transport protein; Pfam 00005: ABC transporter; Pfam 00664: ABC transporter transmembrane region) were employed to identify the PIN, PILS, AUX/LAX and ABCB auxin transporter families of maize. Pfam 01490 was used for AUX/LAX family identification; Pfam 03547 was used for PIN family; Pfam PF03547 was used for PILS family; Pfam 00005 and Pfam 00664 were used for ABCB family. These five profiles were used to search the complete proteome of maize available in phytozome (http://www.phytozome.net/). All the obtained sequences were sorted as unique sequences for further membrane transport protein domain search using InterProScan Sequence Search (http://www.ebi.ac.uk/Tools/pfa/iprscan/).

### Phylogenetic tree building, intron/exon structure, genome distribution and motif prediction

Multiple sequence alignments were performed on the PIN, PILS, LAX and ABCB proteins using ClustalW with the default parameters, and the alignments were then adjusted manually. The information of *Arabidopsis* and rice auxin transporter encoding genes were listed in S1 Table. The alignments were visualized subsequently by software GeneDoc (http://www.nrbsc.org/gfx/genedoc/), and a phylogenetic tree was constructed with aligned five ZmLAX protein sequences, fifteen ZmPIN protein sequences, nine ZmPILS protein sequences and thirty-five ZmABCB protein sequences using MEGA5.1 (http://www.megasoftware.net/mega5/mega.html) employing the neighbor-joining (NJ) method. The coding sequences (CDS) were obtained from the maize sequencing database. Exon-intron organizations of *ZmPIN*, *ZmPILS*, *ZmLAX* and *ZmABCB* genes were identified by comparing the coding sequences with their corresponding genomic sequences using Gene Structure Display Server (GSDS) software (http://gsds.cbi.pku.edu.cn/). We drew a map of the distribution of *ZmPIN*, *ZmPILS*, *ZmLAX* and *ZmABCB* genes throughout the maize genome.

### Transmembrane helices structure predictions

The transmembrane domains were estimated using TMHMM2: http://www.cbs.dtu.dk/services/TMHMM/. The data of all ZmLAX, ZmPIN, ZmPILS and ZmABCB proteins were listed in S1 Fig.

### Expression analysis of maize auxin transporter genes

Spatial-temporal expression pattern analysis of *ZmLAXs*, *ZmPINs*, *ZmPILSs* and *ZmABCBs* using microarray data for sixty types of tissues and organs housed in the Bio-Array Resource for Plant Biology at the Maize Genetics and Genomics Database (MaizeGDB: http://www.maizegdb.org/expression/) were used. To confirm the microarray data, we chosen four representational tissues and organs including leaves, roots and shoots from two-week seedlings and flowers from two-month plants to test the expression levels of these auxin transporter genes by qRT-PCR methods. The database contains expression as reported by Sekhon et al 2011 mapped to B73 RefGen_v2 [39]. The methods utilized for normalization and to adjust background, as well as detection calls, P-value calculation and adjustment have been described previously [39]. The raw data from the MaizeGDB about these auxin transporter genes is listed in S2 Table.

### RNA isolation and Quantitative RT-PCR

The methods, including RNA extraction from various tissues, reverse transcription and qRT-PCR, were performed according to Shen’s work [33]. Total RNA from different tissues or organs including cotyledons, leaves, roots, shoots and flowers were extracted using a Plant RNeasy Mini kit (Qiagen) according to the manufacturer’s instruction. DNase I was used to remove any genomic DNA contamination from total RNA. The primers sequences of qRT-PCR are listed in S3 Table. In the experiment of tissues-specific expression analysis, the gene *Zm-Actin* and *18S rRNA* gene were used as internal standards to calculate relative fold differences basing on the comparative cycle threshold (2^-ΔΔ*Ct*^) values. The primer sequences of Actin gene were up- CACCTTCTACAACGAGCTCC/dn-CAGTCAGGATCTTCATGAGG and the primer sequences of *18S rRNA* gene were up-AGTTTGAGGCAATAACAGGTCT /dn-GATGAAATTTCCCAAGATTACC. Heat map representation was performed using the average *Ct* value with Treeview 1.6 software to visualize the tissues-specific expression analysis data. The logarithm of expression level compared to *ZmACTIN*/10000 or *18S rRNA*/10000 were used by Treeview to visualize as heat map. The expression levels of *ZmACTIN* or *18S rRNA* genes were defined as log (10000) = 4. Histograms were used to show the data of abiotic stress response experiments. The expression levels of *ZmPIN*, *ZmPILS*, *ZmLAX* and *ZmABCB* genes under mock treatment were defined as 1. All the expression analysis was carried out for five biological repeats and the values shown in figures represent the average values of these five repeats.

### Analysis of auxin and stress-related *cis*-elements

The promoters (-1500 to -1 bp before ATG) of *ZmLAX*, *ZmPIN*, *ZmPILS* and *ZmABCB* genes were scanned for auxin and stress-related *cis*-elements. The sequences data of *ZmLAX*, *ZmPIN*, *ZmPILS* and *ZmABCB* promoters were obtained from phytozome 10.1. Nine *cis*-elements were used in this study: dehydration and cold response (DRE/CRT), ABA responsive element (ABRE), ARF1 binding site (AuxRE), SA-responsive promoter element (SARE), environmental signal response (G-box), WRKY binding site (W-box), CAMTA binding site (CG-box), PHR1 binding site (P1BS) and sulfur-responsive element (SURE).

## Results

### Genome-wide identification of *PIN*, *LAX* and *ABCB* genes in maize

In our study, we used the previously reported PIN, PILS, AUX/LAX and ABCB proteins from *Arabidopsis* as BLAST queries to search the public genomic database (http://www.phytozome.net/). The hidden Markov model (HMM) profiles (Pfam 01490: transmembrane amino acid transporter protein; Pfam 03547: membrane transport protein; Pfam 00005: ABC transporter; Pfam 00664: ABC transporter transmembrane region) were employed to identify the ZmLAX, ZmPIN, ZmPILS and ZmABCB protein families. A total of five *ZmLAX* genes, 15 *ZmPIN* genes, nine *ZmPILS* genes and 35 *ZmABCB* genes were identified after comprehensive search. The protein and CDS sequences of these genes were subsequently downloaded. Information on 55 auxin transporter encoding genes, such as: names, locus ID, open reading frame (ORF) lengths, intron/exon numbers, chromosome locations and basic deduced polypeptide parameters, are listed in Table 1.

**Table 1:** Auxin transport genes in maize.

Gene	Locus ID	ORF length (bp)	No. of introns	Chr No.	Chr location	Deduced polypeptide		
						Length (aa)	Mol wt (kDa)	pI
*ZmLAX1*	GRMZM2G149481	1560	7	1	33317543–33321538	520	57.98	8.97
*ZmLAX2*	GRMZM2G129413	1710	2	1	119652826–119656777	570	62.74	8.79
*ZmLAX3*	GRMZM2G127949	1470	7	3	178002561–178009059	490	54.46	8.39
*ZmLAX4*	GRMZM2G045057	1455	5	4	204125069–204127812	485	53.87	8.67
*ZmLAX5*	GRMZM2G067022	1953	6	6	148967914–148977647	651	72.48	10.1
*ZmABCB1*	GRMZM5G820122	3123	3	1	9303132–9306911	1041	114.49	8.51
*ZmABCB2*	GRMZM2G401769	2367	9	1	42186629–42192907	789	86.14	4.98
*ZmABCB3*	GRMZM2G032936	1362	14	1	54843018–54851106	454	50.07	5.57
*ZmABCB4*	GRMZM2G315375	4137	4	1	202298103–202305287	1379	148.11	8.72
*ZmABCB5*	GRMZM2G084181	4881	27	2	9114721–9126810	1627	183.15	6.75
*ZmABCB6*	GRMZM2G072850	3798	8	2	6171190–6178089	1266	137.26	8.06
*ZmABCB7*	GRMZM2G032218	1537	10	2	13856499–13864605	1540	172.91	8.1
*ZmABCB8*	GRMZM2G388539	1740	8	2	56923761–56929497	580	63.13	8.61
*ZmABCB9*	GRMZM2G365957	3684	5	2	103336614–103342858	1288	134.28	5.95
*ZmABCB10*	GRMZM2G167658	4551	4	2	109104216–109109170	1517	162.38	9.1
*ZmABCB11*	GRMZM2G119894	3987	11	3	50093167–50099618	1329	143.17	8.59
*ZmABCB12*	GRMZM2G049351	1785	5	3	144080317–144083641	595	64.47	5.18
*ZmABCB13*	GRMZM2G025860	3693	8	3	201622569–201629041	1231	134.38	8.46
*ZmABCB14*	GRMZM2G086730	3879	11	3	227824114–227836467	1293	139.15	8.04
*ZmABCB15*	GRMZM2G441722	3591	10	4	33816544–33821596	1197	130.75	5.8
*ZmABCB16*	GRMZM2G004748	3786	9	4	157609283–157616979	1262	138.43	9.22
*ZmABCB17*	GRMZM2G146034	2418	2	4	234421148–234429271	806	87.64	7.72
*ZmABCB18*	GRMZM2G072071	1944	17	5	6877441–6883253	648	69.92	8.01
*ZmABCB19*	GRMZM5G843192	3768	5	5	172023958–172028243	1256	135.16	7.99
*ZmABCB20*	GRMZM5G832772	3867	9	7	123359731–123389161	1289	143.61	6.29
*ZmABCB21*	GRMZM2G142870	4527	12	8	45260943–45275982	1509	165.75	7.29
*ZmABCB22*	GRMZM2G082385	3807	11	8	64497963–64503845	1269	136.07	6.13
*ZmABCB23*	GRMZM2G153961	1164	2	8	64405469–64406946	388	41.81	5.28
*ZmABCB24*	GRMZM5G843537	2295	7	8	64419486–64423645	765	82.8	8.35
*ZmABCB25*	GRMZM2G014089	3234	10	8	152715030–152719617	1078	116.01	8.39
*ZmABC26*	GRMZM5G874756	1857	1	9	6998377–7000813	618	65.89	8.92
*ZmABCB27*	GRMZM2G081573	2379	21	9	12828239–12877329	793	88.85	8.51
*ZmABCB28*	GRMZM2G111903	4440	12	9	17739642–17751534	1480	163.45	7.98
*ZmABCB29*	GRMZM2G113203	4431	10	9	57011529–57017762	1477	162.16	8.02
*ZmABCB30*	GRMZM5G891159	4239	10	9	138972581–138980123	1413	155.81	6.18
*ZmABCB31*	GRMZM2G361256	3984	9	9	151361774–151369052	1328	146.14	6.78
*ZmABCB32*	GRMZM2G333183	3720	9	10	80571279–80577409	1240	136.53	8.44
*ZmABCB33*	GRMZM2G111462	3909	6	10	125844982–125849427	1303	141.62	8.54
*ZmABCB34*	GRMZM2G413774	3969	10	10	135364950–135393105	1323	148.22	8
*ZmABCB35*	GRMZM2G085236	3798	8	10	144913379–144921010	1266	137.41	8.19
*ZmPIN5b*	GRMZM2G148648	792	4	1	193455354–193457161	264	27.64	6.96
*ZmPIN5c*	GRMZM2G040911	1095	3	2	191860487–191864149	365	38.43	9.07
*ZmPIN13*	GRMZM2G064941	891	0	2	212649453–212650346	297	31.84	11.51
*ZmPIN14*	GRMZM2G471745	816	0	2	212664065–212664888	272	29.42	11.79
*ZmPIN5a*	GRMZM2G025742	1146	/	3	160753018–160757148	382	40.57	9.17
*ZmPIN9*	GRMZM5G859099	1299	4	3	187809760–187812845	433	46.84	7.3
*ZmPIN8*	GRMZM5G839411	1086	4	3	202930545–202933072	362	39.94	8.87
*ZmPIN10a*	GRMZM2G126260	2058	5	3	214899506–214904595	686	73.57	9.38
*ZmPIN1c*	GRMZM2G149184	1791	5	4	182007439–182010555	597	64.65	8.16
*ZmPIN1d*	GRMZM2G171702	1740	5	4	186642791–186645569	580	61.05	9.11
*ZmPIN5d*	GRMZM2G175983	828	0	4	163010632–163012173	276	29.9	9.42
*ZmPIN15*	GRMZM2G021364	2238	13	5	152572571–152581759	746	81.26	9.32
*ZmPIN1b*	GRMZM2G074267	1785	5	5	206727149–206730565	595	64.51	8.9
*ZmPIN1a*	GRMZM2G098643	1803	5	9	3650766–3654174	601	65.19	8.77
*ZmPIN10b*	GRMZM2G160496	1743	5	9	16725140–16727538	581	61.84	8.86
*ZmPILS1*	GRMZM2G070563	927	6	2	216298100.216300491	309	34.58	5.83
*ZmPILS2*	GRMZM2G331322	435	5	2	226922493.226924568	145	15.81	6.95
*ZmPILS3*	GRMZM2G112598	1017	5	3	29620546.29623590	339	36.8	5.9
*ZmPILS4*	GRMZM2G050089	1299	10	3	185812406.185817191	433	46.75	8.25
*ZmPILS5*	GRMZM2G030125	1353	1	4	135339533.135342026	451	49.43	6.64
*ZmPILS6*	GRMZM2G475148	1731	7	7	115242111.115247553	577	63.65	8.81
*ZmPILS7*	GRMZM2G043254	1362	9	7	133486108.133489711	454	48.86	8.85
*ZmPILS8*	GRMZM2G072632	1101	7	10	90297677.90299717	367	40.29	7.16
*ZmPILS9*	GRMZM2G007481	1362	9	10	128473459.128477062	454	48.89	8.85

The sizes of the deduced ZmLAX proteins varied slightly ranging from 485 amino acids (ZmLAX4) to 651 amino acids (ZmLAX5), the corresponding molecular masses varied from 53.87 kDa to 72.48 kDa, and the predicted isoelectric points varied from 8.39 (ZmLAX3) to 10.10 (ZmLAX5). The sizes of the deduced ZmPIN proteins varied evidently ranging from 264 amino acids (ZmPIN5b) to 746 amino acids (ZmPIN15), the corresponding molecular masses varied from 27.64 kDa to 81.26 kDa, and the predicted isoelectric points varied from 6.96 (ZmPIN5b) to 11.79 (ZmPIN14). The sizes of the deduced ZmPILS proteins varied greatly ranging from 435 amino acids (ZmPILS2) to 1731 amino acids (ZmPILS6), the corresponding molecular masses varies from 15.81 kDa to 63.65 kDa, and the predicted isoelectric points varied from 5.83 (ZmPILS1) to 8.85 (ZmPILS9). The sizes of the deduced ZmABCB proteins varied largely ranging from 388 amino acids (ZmABCB23) to 1540 amino acids (ZmABCB7), the corresponding molecular masses varied from 41.81 kDa to 172.91 kDa, and the predicted isoelectric points varied widely from 4.98 (ZmABCB2) to 9.22 (ZmABCB16). The data suggested that different auxin transporter proteins might function in auxin relocation when plants were exposed to different microenvironments.

### Chromosomal distribution and expansion patterns of Z*mPIN*, *ZmPILS*, *ZmLAX* and *ZmABCB* genes

**Fig 1:**
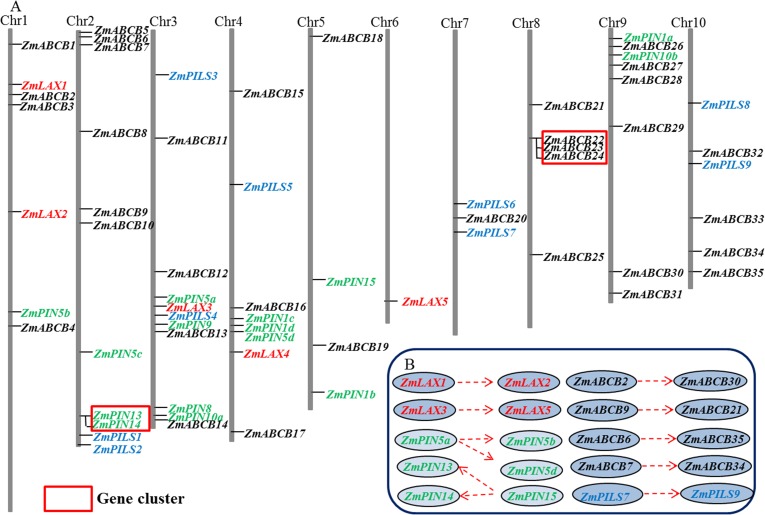
Chromosomal distribution and expansion patterns of maize auxin transporter encoding genes. (A) The genome visualization tool SyMAP Synteny Browser was employed (http://www.symapdb.org/) to analyze the maize genome. Maize chromosomes were arranged in blocks. Fifteen *ZmPIN* genes, nine *ZmPILS* genes, five *ZmLAX* genes and thirty-five *ZmABCB* genes were among ten chromosomes. *ZmPIN*, *ZmPILS*, *ZmLAX and ZmABCB* family genes are mapped by locus and the gene clusters were indicated by red boxes. (B) Gene segmental duplications were showed with dotted arrows.

Based on the start position of each maize *LAX*, *PIN*, *PILS* and *ABCB* gene on the chromosomes, we mapped all five *ZmLAX* genes, 15 *ZmPIN* genes, nine *ZmPILS* genes and 35 *ZmABCB* genes on ten chromosomes unevenly (Fig. 1A, Table 1). Chromosomes 2 and 3 contained the largest number of auxin transporter encoding genes (nine genes in each chromosome), but chromosome 6 only contained one gene. In the monocot, *Sorghum bicolor*, and the dicot, *Arabidopsis*, many of the auxin transporter encoding genes were clustered [27,33]. According to the definition of gene clusters [40], only two small gene clusters were identified. The first gene cluster consisted of two *ZmPIN* genes (*ZmPIN13* and *ZmPIN14*) and the second gene cluster consisted of three *ZmABCB* genes (*ZmABCB22*, *ZmABCB23* and *ZmABCB24*) (Fig. 1A).

Gene duplication events, including tandem and segmental duplications, are the main contributors to evolutionary momentum [41,42]. *ZmLAX*, *ZmPIN*, *ZmPILS* and *ZmABCB* family duplication modes throughout the maize genome were analyzed to uncover the genetic relationships between the different genes. Two segmental duplications occurred in *ZmLAX* gene family: *ZmLAX1*/*ZmLAX2* and *ZmLAX3*/*ZmLAX5*. Interestingly, our data suggested that Z*mPIN5a* and *ZmPIN15* may function as ancestral genes during the evolutionary process. Segmental duplications also occurred between *ZmPIN5a* and *ZmPIN5b/ZmPIN5d*, and another two segmental duplications occurred between *ZmPIN15* and *ZmPIN13*/*ZmPIN14*. There was tandem duplication between *ZmPIN13* and *ZmPIN14*. One segmental duplication occurred in the *ZmPILS* gene family: *ZmPILS7*/*ZmPILS9*, but four segmental duplications were found in the *ZmABCB* gene family: *ZmABCB2*/*ZmABCB30*, *ZmABCB9*/*ZmABCB21*, *ZmABCB6*/*ZmABCB35* and *ZmABCB7*/*ZmABCB34* (Fig. 1B).

### ZmPIN, ZmPILS, ZmLAX and ZmABCB protein structure analysis

Fifteen ZmPIN proteins have been found in maize and most of them have a classical conservative domain structure, which consists of two hydrophobic domains (V1 and V2) and one hydrophilic loop containing three conserved regions (C1-C3) [43]. The ZmPIN14 and ZmPIN5a proteins only contained one hydrophobic loop and lacked the V1 and V2 regions. Most ZmPIN proteins contained nine or ten transmembrane helices. However, ZmPIN14 and ZmPIN5a only had two transmembrane helices, and ZmPIN15 had three (S1 Fig.). The ZmPILS proteins shared similar conservative domain structure with ZmPIN proteins, and the ZmPILS1 and ZmPILS2 proteins only had two transmembrane helices. All five ZmLAX proteins contained a highly conserved core region, which was composed of ten transmembrane helices. Amino acid composition analysis indicated that C-terminus of the ZmLAX was proline-rich and the N-terminus of ZmLAX was acidic amino acids-rich. Previous studied have shown that the ABCB family was a subgroup of the ABC transporter superfamily [44]. Multiple sequence alignment showed that most ZmABCB proteins contained a universal structure with a nucleotide binding domain and a transmembrane domain. These two domains were separated by a less conserved linker loop. However, some ZmABCB proteins only contained a nonconservative transmembrane domain, such as: ZmABCB3, ZmABCB8, ZmABCB13, ZmABCB17, ZmABCB18, ZmABCB23 and ZmABCB28 (S1 Fig.).

### Phylogenetic analysis of the *LAX*, *PIN*, *PILS* and *ABCB* family genes

**Fig 2:**
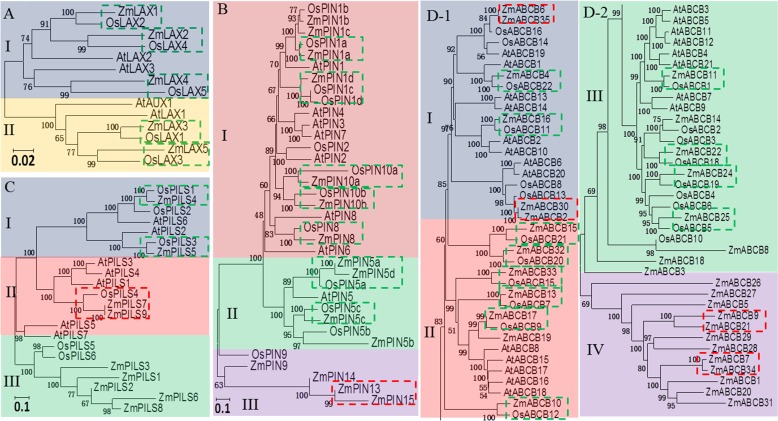
Phylogenetic relationship analysis of auxin transporter family between maize, *Arabidopsis* and rice. Bootstrap values are presented for all branches. (A) LAX protein family: inventory of AtLAX and OsLAX families is based on TAIR and TIGR rice databases. (B) PIN protein family: sequence data on AtPIN and OsPIN families is based on TAIR annotation and Shen’s publish. (C) PILS protein families: inventory of AtPILS and OsPILS families is listed at S1 Table. (D) ABCB protein family: inventory of AtABCB and OsABCB families is based on the ABC superfamily review by Verrier *et al*. Different colors indicated different subfamilies. The ortholog genes between maize and *Arabidopsis* or rice were indicated by green dotted boxes. The paralog genes within maize were indicated by red dotted boxes.

A number of studies have revealed the biological functions of the four major auxin transporter encoding gene families in different plant species [30,31,45,46]. Investigation of the evolutionary relationships among maize, *Arabidopsis* and rice helps us to understand the possible roles in these auxin transporter encoding genes in maize. Some background information on the *Arabidopsis* and rice auxin transporter genes is listed in S1 Table. Our data showed that the *LAX* family genes were divided into two subfamilies (I and II). The phylogenetic tree showed that some ortholog genes in maize and rice were more closely related than their equivalents in maize and *Arabidopsis*. Five ortholog gene pairs existed between the maize and rice *LAX* gene families: *ZmLAX1*/*OsLAX2*, *ZmLAX2*/*OsLAX4*, *ZmLAX4*/*OsLAX5*, *ZmLAX3*/*OsLAX1* and *ZmLAX5*/*OsLAX3*. All the *PIN* family genes could be classified into three subfamilies (I, II and III), and four *PIN* ortholog gene pairs were found between maize and rice: *ZmPIN1a*/*OsPIN1a*, *ZmPIN1d*/*OsPIN1c*/*OsPIN1d*, *ZmPIN10a*/*OsPIN10a*, *ZmPIN10b*/*OsPIN10b*, *ZmPIN5a*/*ZmPIN5d*/*OsPIN5a*, *ZmPIN8*/*OsPIN8* and *ZmPIN5c*/*OsPIN5c*. One paralog gene pair occurred in the maize *PIN* gene family: *ZmPIN13*/*ZmPIN15*. The PILS proteins were also grouped into three subfamilies (I, II and III). Three *PILS* ortholog gene pairs have been found between maize and rice: *ZmPILS4*/*OsPILS1*, *ZmPILS5*/*OsPILS3* and *ZmPILS7*/*ZmPILS8*/*OsPILS4*. The *ABCB* genes were grouped into four subfamilies (I, II, III and IV). Twelve *ABCB* ortholog gene pairs were found between maize and rice: *ZmABCB4*/*OsABCB22*, *ZmABCB16*/*OsABCB11*, *ZmABCB15*/*OsABCB21*, *ZmABCB32*/*OsABCB20*, *ZmABCB33*/O*sABCB15*, *ZmABCB13*/*OsABCB7*, *ZmABCB17*/*OsABCB9*, *ZmABCB10*/*OsABCB12*, *ZmABCB11*/*OsABCB1*, *ZmABCB22*/*OsABCB18*, *ZmABCB24*/*OsABCB19* and *ZmABCB25*/*OsABCB5*. Totally, four paralog gene pairs existed in maize *ABCB* gene family: *ZmABCB6*/*ZmABCB35*, *ZmABCB30*/*ZmABCB2*, *ZmABCB9*/*ZmABCB21* and *ZmABCB7*/*ZmABCB34* (Fig. 2).

### Tissue-specific expression pattern and exon–intron structure analysis of *ZmPIN*, *ZmPILS*, *ZmLAX* and *ZmABCB*

The previously reported the involvement of these auxin transporter encoding genes in the control of the auxin influx and efflux polar transport prompted us to investigate the expression levels of *ZmPIN*, *ZmPILS*, *ZmLAX* and *ZmABCB* genes in different tissues and organs. The microarray data of 60 types of different tissues and organs housed in the MaizeGDB database were used to analyze the spatial-temporal expression pattern of *ZmLAX*, *ZmPIN*, *ZmPILS* and *ZmABCB* genes. The results showed that most of the auxin transporter genes in maize had tissue-specific expression patterns (S2 Fig.). To confirm the spatio expression dynamics, qRT-PCR was used to monitor the transcript accumulations of *ZmPIN*, *ZmPILS*, *ZmLAX* and *ZmABCB* genes in four representational organs. These were the leaves, roots and shoots from 2-week old seedlings and flowers from 2-month old plants.

**Fig 3:**
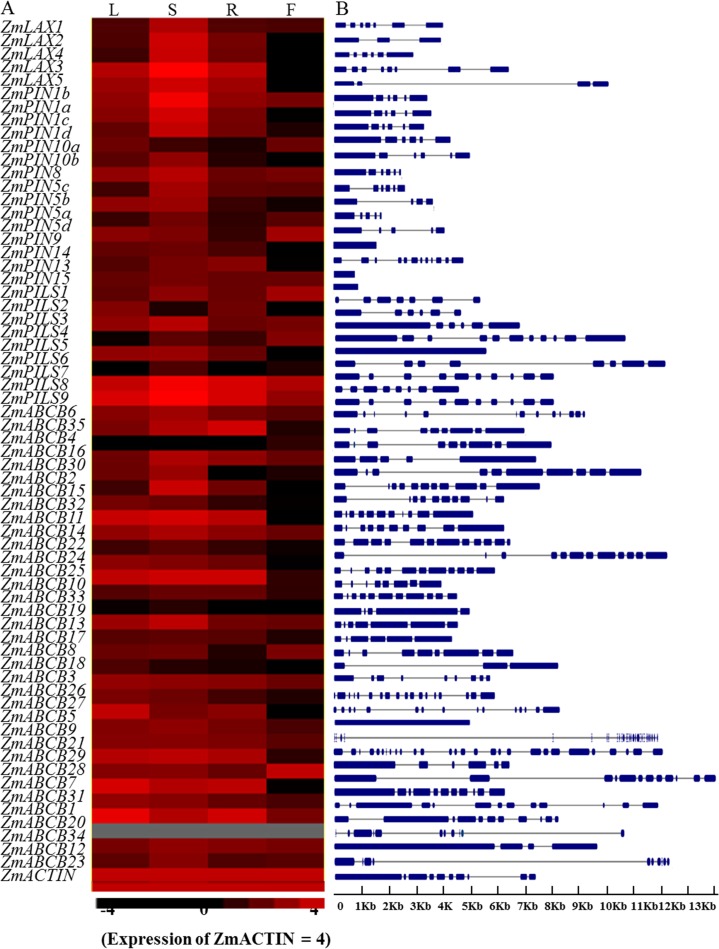
Tissues-specific expressions and exon-intron structures of *ZmLAX*, *ZmPIN*, *ZmPILS* and *ZmABCB* genes. (A) Tissues-specific expression patterns of *ZmLAX*, *ZmPIN*, *ZmPILS* and *ZmABCB* genes. L: leaf; R: root; S: shoot; F: flower. Levels of different colors were shown on a log scale from the high to the low expression of each *ZmPIN*, *ZmPILS*, *ZmLAX* and *ZmABCB* gene. The expression level of *ZmACTIN* or *18S RNA* gene were defined as log (10000) = 4. (B) Exon-intron structure analysis of *ZmLAX*, *ZmPIN*, *ZmPILS* and *ZmABCB* genes. The exons were indicated by blue boxes; the introns were indicated by gray lines.

Most transcripts from the *ZmLAX*, *ZmPIN*, *ZmPILS* and *ZmABCB* family genes increased in the selected tissues, except for *ZmABCB34* (Fig. 3A), whose expression level could not be detected in any of the tissues and organs. A majority of the *ZmPIN*, *ZmPILS*, *ZmLAX* and *ZmABCB* genes displayed distinct tissue-specific expression patterns across the four tissues and organs. Our data showed that the *ZmLAX* gene family transcript abundance was highest in the shoots and lowest in flowers, and that *ZmPIN1b*, *ZmPIN5a*, *ZmPIN10a*, *ZmPIN13*, *ZmPIN14* and *ZmPIN15* were present in all representative tissues and organs. However, the rest of *ZmPIN* genes were more weakly expressed in flowers than in the other organs, except for *ZmPIN10a* and *ZmPIN1d*, which showed the lowest expression in roots; and *ZmPIN5b* and *ZmPIN8*, which showed the lowest expression in leaves. Most of the *ZmPIN* genes were preferentially expressed in the shoots. In the *ZmPILS* gene family, *ZmPILS8* and *ZmPILS9* were much more highly expressed than the other *ZmPILS* genes. Within a similar way to the *ZmPIN* and *ZmLAX* gene family, *ZmABCB* gene expression was much higher in the roots, shoots and leaves than in the flowers. The qRT-PCR values of the *ZmPIN*, *ZmPILS*, *ZmLAX* and *ZmABCB* gene expression levels are listed in S4 Table.

The exon–intron structures of the *ZmPIN*, *ZmPILS*, *ZmLAX* and *ZmABCB* family genes were highly diversified. The *ZmLAX* family gene exon numbers varied from three (*ZmLAX2*) to eight (*ZmLAX3*). Some *ZmPIN* family genes had similar gene exon-intron organizations, such as *ZmPIN1c*, *ZmPIN1d*, *ZmPIN8* and *ZmPIN1a*, *ZmPIN1b*, *ZmPIN10a*. Interestingly, three of the *ZmPIN* family genes (*ZmPIN13*, *14* and *5b*) only contained one exon and no introns. In the *ZmPILS* gene family, *ZmPILS5* also contained one exon and no introns. The exon-intron organization of the *ZmABCB* genes varied considerably in terms of intron numbers and intron phase (Fig. 3B). This suggested that the *ZmABCB* gene structures were shuffled during the evolutionary process [47].

### *ZmLAX*, *ZmPIN*, *ZmPILS* and *ZmABCB* gene expression regulation by auxin

Auxin transporters regulate auxin relocation during plant growth and development [48]. Exogenous IAA stimulation accelerates or blocks the endogenous auxin transport between different organs [33,49]. In order to gain a better understanding of how auxin transporter encoding genes are involved in responses to exogenous hormones, we analyzed the expression profiles of *ZmLAX*, *ZmPIN*, *ZmPILS* and *ZmABCB* genes under 10 μM IAA for 48 hours in the shoots and roots. Total RNA isolated from the shoots and roots of IAA-treated seedlings and control seedlings was subjected to qRT-PCR analysis at different time points (0, 12, 24 and 48 hours).

**Fig 4:**
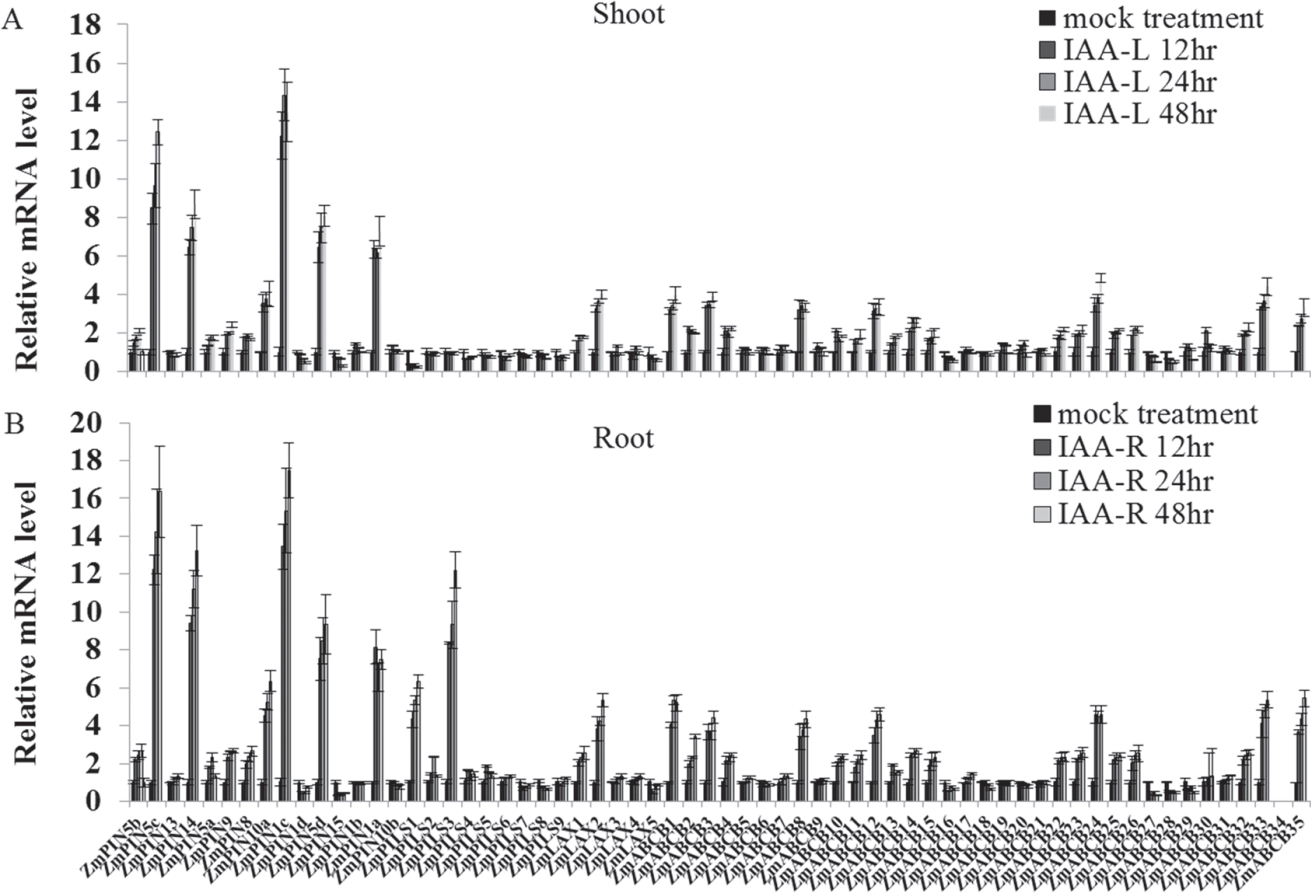
Real-time quantitative RT-PCR (qRT-PCR) analysis of *ZmLAX*, *ZmPIN*, *ZmPILS* and *ZmABCB* genes in plants under IAA treatment in both shoots (A) and roots (B). Total RNA was extracted from the seedlings shoots and roots of maize for expression analysis. The histogram shows the relative expression levels of *ZmLAX*, *ZmPIN*, *ZmPILS* and *ZmABCB* genes under IAA treatment (10 μM, 48 hours) compared to the mock expression level. Untreated seedlings were used as mock seedlings. The relative mRNA level of individual genes was normalized with respect to the *ZmACTIN* gene (defines as 1). The data were analyzed by five independent repeats, and standard deviations were shown with error bars.

Our qRT-PCR data indicated that most *ZmPIN*, *ZmPILS*, *ZmLAX* and *ZmABCB* genes were IAA-responsive genes. The expression levels of these genes under IAA treatment are shown in Fig. 4. The expression change trends for these auxin transporter genes over the 48 hours of IAA treatment were similar. IAA treatment increased *ZmPIN5c*, *ZmPIN14*, *ZmPIN1c*, *ZmPIN5d* and *ZmPIN1a* expression levels in the shoots five-fold. In contrast, *ZmPIN1d*, *ZmPIN15*, *ZmLAX5*, *ZmABCB16* and *ZmABCB27-29* expression levels in the shoots were sharply reduced by IAA treatment (Fig. 4A). In the roots, IAA treatment up-regulated the *ZmPIN5c*, *ZmPIN1c*, *ZmPIN1a*, *ZmPILS3*, *ZmABCB4* and *ZmABCB19* expression levels more than five-fold. However, many genes, such as *ZmPIN5b*, *5d*, *9*, *13*, 1*4*, and *15*; *ZmPILS7* and *ZmPILS8*; *ZmLAX4*; *ZmABCB6*, *7*, *9*, *10*, *12*, *13* and *24*, were considerably down-regulated in the roots by the IAA treatment (Fig. 4B).

### Stress-related *cis*-elements in the *ZmLAX*, *ZmPIN*, *ZmPILS* and *ZmABCB* gene promoters

Transcription factor binds to a specific motif that was called *cis*-element to activate gene transcription in plants [50]. Several *cis*-elements that are involved in stress responses have been well identified in plants, including dehydration and Cold response (DRE/CRT: RCCGAC) [51], ABA responsive element (ABRE: YACGTGK) [52], ARFs binding site (AuxRE: TGTCTC) [53], SA-responsive promoter element (SARE: TGACG) [54], environmental signal response (G-box: CACGTG) [55], WRKY binding site (W-box: TTGACY) [56], CAMTA binding site (CG-box: VCGCGB) [57], PHR1 binding site (P1BS: GNATATNC) [58] and sulfur-responsive element (SURE: GAGAC) [59]. Here, we scan the *ZmPIN*, *ZmPILS*, *ZmLAX* and *ZmABCB* gene -1500bp upstream promoter regions with nine stress-related *cis*-elements to gain clues on how the these gene expressions are responsive to stresses stimuli.

The stress-related motifs were obviously enriched in the promoter regions of *ZmPIN*, *ZmPILS*, *ZmLAX* and *ZmABCB* genes. The numbers of stress-related *cis*-elements in the upstream 1.5 kb regions of *ZmPIN*, *ZmPILS*, *ZmLAX* and *ZmABCB* family genes were listed in S5 Table.

### Expression analysis of *ZmLAX*, *ZmPIN*, *ZmPILS* and *ZmABCB* genes in response to salt, drought and cold treatment

In order to investigate the potential roles of *ZmPIN*, *ZmPILS*, *ZmLAX* and *ZmABCB* genes in response to environmental stresses, we analyzed the expression patterns of 64 auxin transporter encoding genes in the shoots and roots under salt (NaCl), drought and cold (4°C) treatment as described in the Materials and Methods section. Untreated seedlings grown in nutrient solution were used as control seedlings.

**Fig 5:**
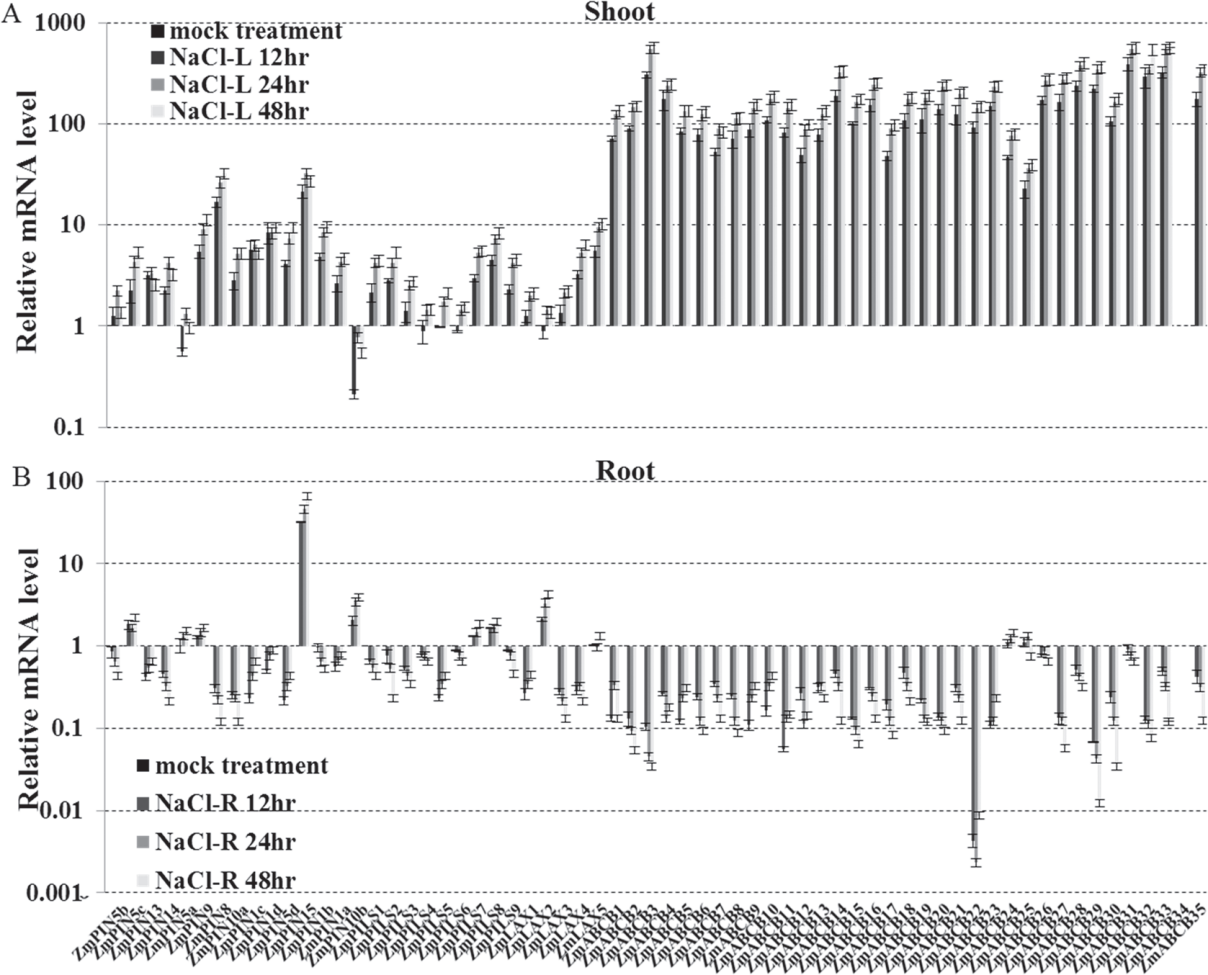
Expression levels of *ZmPIN*, *ZmPILS*, *ZmLAX* and *ZmABCB* gene families in response to salt. Expression levels of *ZmPIN*, *ZmPILS*, *ZmLAX* and *ZmABCB* genes were analyzed by qRT-PCR in both shoot (A) and roots (B) of 14-day-old seedlings, which were treated with 150 mM NaCl (salt) for 48 hours. The relative expression levels were normalized to a value of 1 in mock seedlings. Error bars represent SD from five biological replicates.

**Fig 6:**
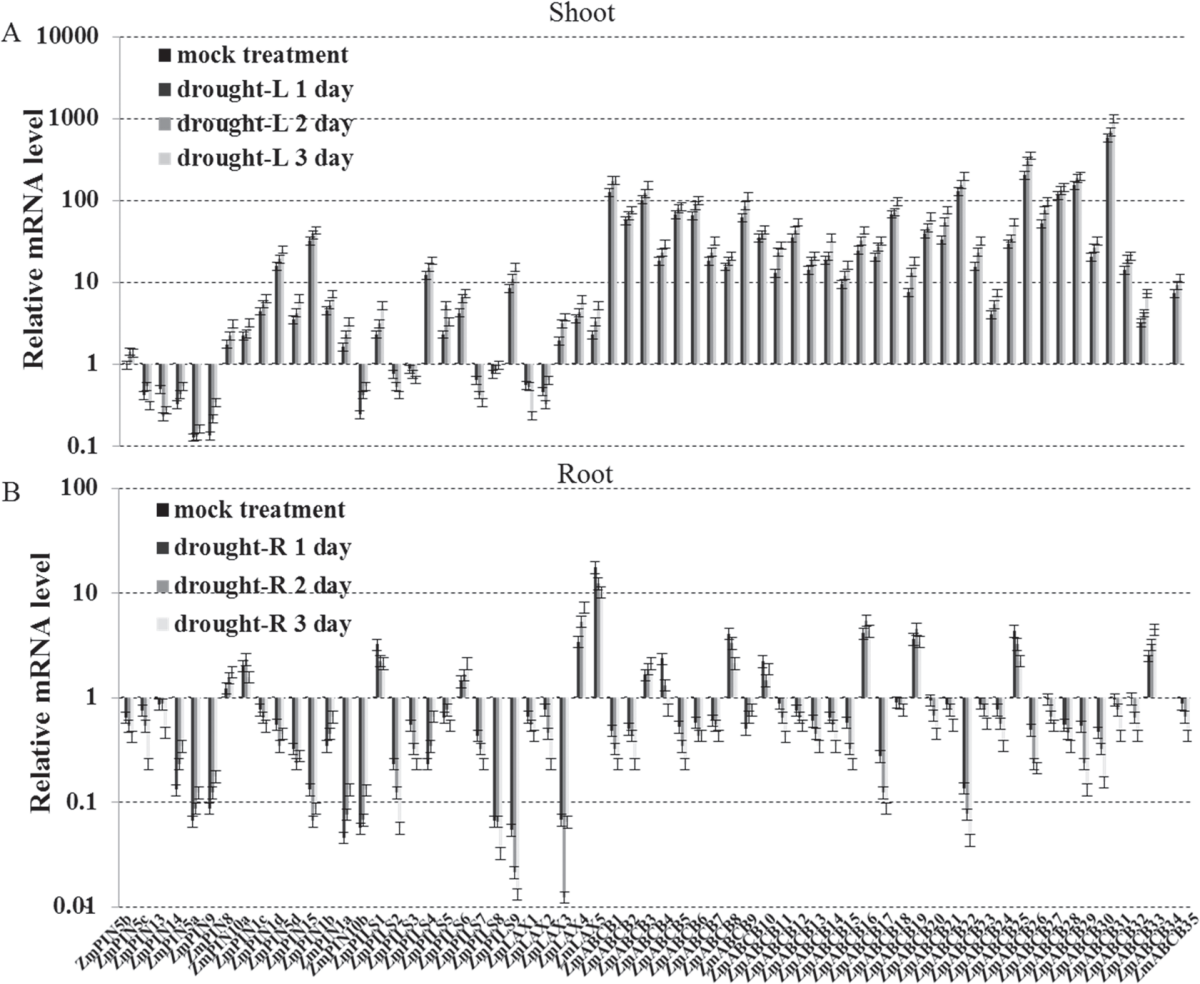
Expression levels of *ZmPIN*, *ZmPILS*, *ZmLAX* and *ZmABCB* gene families in response to drought. Expression levels of *ZmPIN*, *ZmPILS*, *ZmLAX* and *ZmABCB* genes were analyzed by qRT-PCR in both shoot (A) and roots (B) of 14-day-old seedlings, which were treated with dry sand treatment (drought) for 3 days. The relative expression levels were normalized to a value of 1 in mock seedlings. Error bars represent SD from five biological replicates.

The roots and shoots produced different *ZmLAX*, *ZmPIN*, *ZmPILS* and *ZmABCB* expression patterns when they were subjected to the NaCl treatment. Most of the *ZmPIN*, *ZmPILS*, *ZmLAX* and *ZmABCB* genes were up-regulated in the shoots after 48 hours of NaCl treatment. The only exceptions were *ZmPIN5a* and *ZmPIN10b*. However, up-regulation did not generally occur in the roots. Only *ZmPIN5c*, *ZmPIN15*, *ZmPIN10b* and *ZmLAX2* were induced by NaCl treatment in the roots. No significant changes in *ZmPIN5a*, *ZmPIN5b*, *ZmPIN9*, *ZmPIN1b*, *ZmLAX5*, *ZmABCB24-26* and *ZmABCB31* expression levels were detected, and the rest genes were significantly down-regulated in the roots. In a similar manner, most of the *ZmABCB* family genes were also sharply induced (> 100 fold) by NaCl treatment in the shoots, but were significantly down-regulated in the roots (Fig. 5). After a 3-day period of drought treatment, the expression levels of the *ZmABCB* genes, half of the *ZmPIN* and *ZmLAX* genes were up-regulated in shoots. However, the expression levels of most of the *ZmPIN*, *ZmPILS*, *ZmLAX* and *ZmABCB* genes were down-regulated in the roots (Fig. 6).

**Fig 7:**
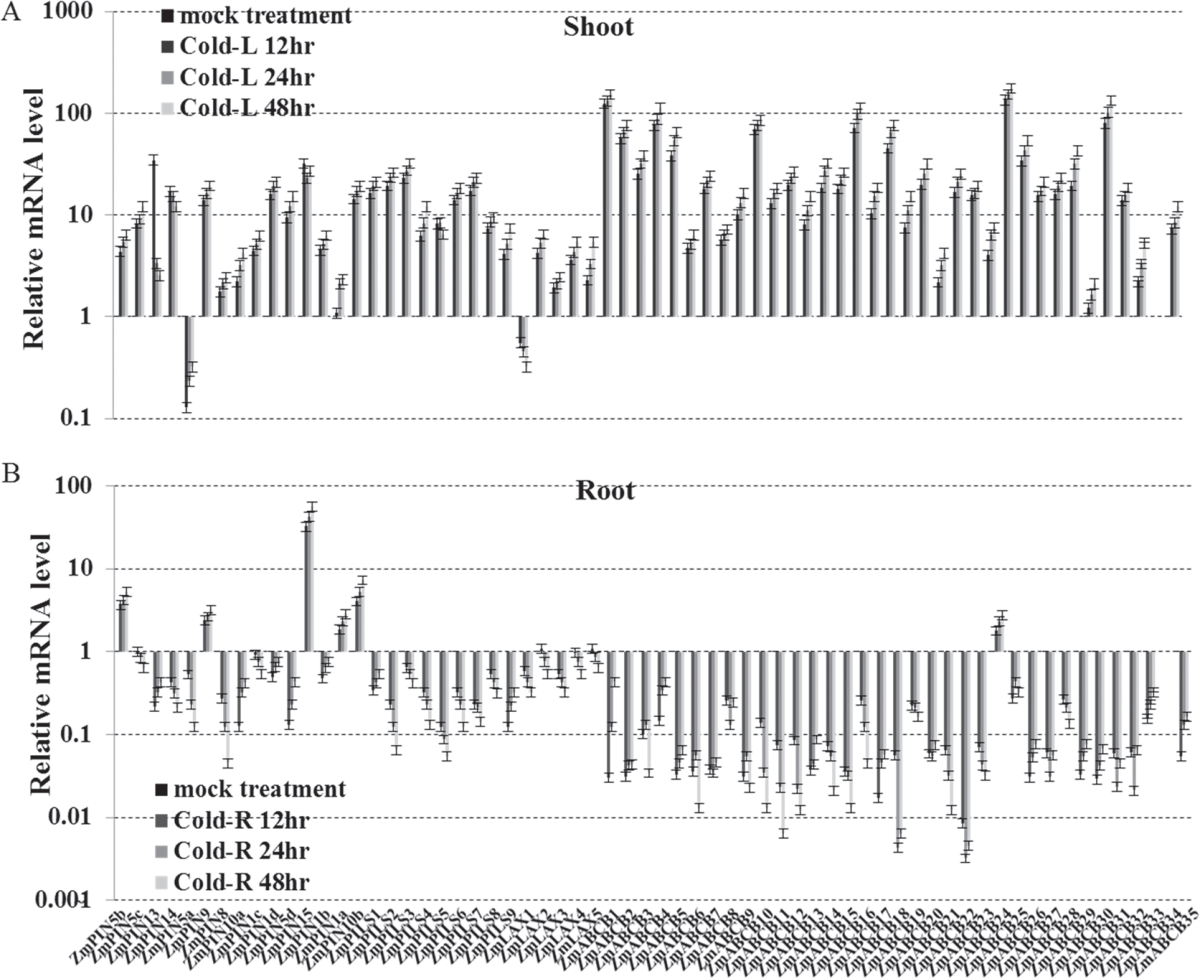
Expression levels of *ZmPIN*, *ZmPILS*, *ZmLAX* and *ZmABCB* gene families in response to cold. Expression levels of *ZmPIN*, *ZmPILS*, *ZmLAX* and *ZmABCB* genes were analyzed by qRT-PCR in both shoot (A) and roots (B) of 14-day-old seedlings, which were treated with cold (4°C) for 48 hours. The relative expression levels were normalized to a value of 1 in mock seedlings. Error bars represent SD from five biological replicates.

Most of the *ZmPIN*, *ZmPILS*, *ZmLAX* and *ZmABCB* genes were induced in the shoots and reduced in the roots by the 4°C treatment after 48 hours. Specially, only *ZmPIN5a* expression was largely reduced in the shoots and only *ZmPIN15* expression was sharply induced expression in the roots (Fig. 7). The auxin transporter encoding genes were responsive to environmental stresses and the shoots and roots showed opposite expression patterns during salt, drought or cold treatment.

## Discussion

Auxin transport plays important roles in maize growth and development, including lateral root initiation and environmental stress resistances [60–62]. Based on their different functions in auxin transport, the auxin transporter encoding genes are divided into four major families. These were *ZmPIN*, *ZmPILS*, *ZmLAX* and *ZmABCB* [9]. In our study, we totally identified 65 auxin transporter encoding genes in maize and focused on the expression profiles of *ZmLAX*, *ZmPIN*, *ZmPILS* and *ZmABCB* genes in order to elucidate how the auxin transporter encoding genes were involved in maize responses to abiotic (salt, drought or cold) stresses.

### Characterization and analysis of the *ZmLAX*, *ZmPIN*, *ZmPILS* and *ZmABCB* gene families in maize

Maize (*Zea mays* L.), a cereal crop with a large genome size (2.3 Gbp), provides food and source of bioethanol worldwide. Our study isolated and characterized the complete *ZmLAX*, *ZmPIN*, *ZmPILS* and *ZmABCB* family genes in maize [63]. The numbers of *LAX*, *PIN* and *PILS* genes in maize were similar to the numbers of these genes in *Arabidopsis*, rice and *sorghum*, but the number of *ABCB* genes in maize was much higher than that in *Arabidopsis*, rice and *sorghum* [33,64]. Genes that were homologous to *Arabidopsis* or rice *LAX*, *PIN*, *PILS* and *ABCB* family genes were present in the maize genome widely. Monocot gene families are always enlarged due to whole genome duplications, which are assumed to occur in the ancestor of monocots about 70 million years ago [25,65]. The relatively high amino acid identities of the LAX, PIN, PILS and ABCB proteins between maize and the model plants rice and *Arabidopsis* suggested that all these auxin transporter encoding genes originated from one or several ancestral sequences [66]. As monocots, the phylogenetic relationship between maize and rice is much closer than the phylogenetic relationship between maize and *Arabidopsis*. Five sister pair genes between maize and rice in the *LAX* family, four in the *PIN* family, three in the *PILS* family and 11 in the *ABCB* family were identified as ortholog genes with bootstrap values ≥99%. However, no highly conserved ortholog gene pairs (bootstrap value ≥99%) between maize and *Arabidopsis* were identified (Fig. 2). Interestingly, several monocot-specific *PIN*, *PILS* and *ABCB* genes were present, according to the phylogenetic analysis. The presence of at least one monocot-specific *PIN* gene has been confirmed by a number of previous studies [24,34,67,68]. The *PIN* genes from subfamily III were all monocot-specific *PINs*, which suggests that the monocot *PIN* family is more divergent than dicot *Arabidopsis PIN* family [49,67]. The *PILS* genes that belonged to subfamily III were also monocot-specific genes. Furthermore, the *ZmABCB* genes that belonged to subfamily IV showed low sequence homology with any *AtABCB* or *OsABCB* genes, and may play specific roles in maize growth and development.

ZmLAX, ZmPIN, ZmPILS and ZmABCB proteins contain several transmembrane helices that are similar to the conserved structure of auxin transporter proteins in *Arabidopsis* and rice [32,49]. The ZmLAX proteins only have one group of membrane-spanning domains, but no variable middle region (S1 Fig.). Most ZmPIN, ZmPILS and ZmABCB proteins contain two groups of membrane-spanning domains in the N- and C-termini, and a highly heterogeneous hydrophilic region, which is located at the center of each protein (S1 Fig.). The PIN protein hydrophilic loop partially modulates the intracellular auxin homeostasis, which is plastic depending on cell type and developmental stage [69]. The presence of the hydrophilic region in maize PIN and ABCB proteins suggested that they had a similar trafficking behavior to the model plants. A combination of phylogenetic and domain structural analyses showed that PIN and ABCB protein functions were conserved between dicots and monocots [70].

### Tissue-specific and auxin response expression pattern analysis of *ZmPIN*, *ZmPILS*, *ZmLAX* and *ZmABCB* family genes

Tissue-specific expression analysis of *ZmPIN*, *ZmPILS*, *ZmLAX* and *ZmABCB* genes showed that all these auxin transporter encoding genes were expressed in the leaves, roots and shoots with different intensity. Plant *PIN*, *PILS*, *LAX* and *ABCB* genes have previously been shown to be involved in growth and development [14,17,27]. The differential expressions of most of the maize *PIN*, *PILS*, *LAX* and *ABCB* genes in different tissues and organs indicated that they were actively involved in regulating growth and development in maize. Specially, most *ZmPIN*, *ZmPILS* and *ZmLAX* genes were highly expressed in the shoots, which suggested that these genes were responsible for long-range auxin transport from the shoot tip to the roots. Previous studies have shown that *ZmPIN1a* and *ZmPIN1b* genes were highly expressed in lateral root caps and *ZmPIN1c* was preferentially expressed in the post-embryonic roots and stems [66]. Our data confirmed that *ZmPIN1* homologous genes were highly expressed in the roots, which suggested that they may take part in maize root architecture determination [24]. Some *ZmPIN* genes also had a developmental stage-specific expression pattern. *ZmPIN1a and ZmPIN1b* were always expressed in the lateral developing primordia of the shoot apical meristem, tassels, ears and in the inner core of the meristems [24,71]. *ZmLAX1*, a close maize homolog of *AtAUX1*, showed expression in the tips of primary roots and leaf primordia [72]. However, a further investigation was required to uncover that how these auxin transporter genes were participated in the development regulation under different developmental stages.

Auxin plays important roles in various developmental processes and its transport between different tissues is mediated by influx (AUX/LAX and ABCB) and efflux (PIN, PILS and ABCB) carriers [73]. To determine whether these auxin transporter encoding genes were also involved in phytohormone signaling, we analyzed expression profiles of these auxin transporter gene families under various phytohormone treatments. In *Arabidopsis*, *AtPIN6* is expressed in specific cell and tissue types, and can be induced by auxin by repressive chromatin modification. [74]. The auxin influx genes, *AtLAX1* and *AtLAX3*, were induced obviously in the roots after auxin treatment [75]. *AtABCB4* is up-regulated by 2,4-D application [76] and *AtABCB1*, which is located in the shoot and root apices, is also induced by IAA treatment [77]. In rice, *OsABCB14*, which is involved in auxin transport and iron homeostasis, also rapidly responded to exogenous auxin [31]. In maize, *ZmABCB35* (GRMZM2G085236), present closest sequence similarity to *OsABCB14* [70], may be also involved in iron uptake and homeostasis. *OsPIN1a* expression showed a five-fold increase after IAA treatment [32]. Some maize auxin transporter encoding genes were also found to be responsive to auxin stimuli in both the roots and shoots, which is similar to the responses in *Arabidopsis* and rice. The *OsPIN1a* ortholog gene in maize, *ZmPIN1a* and *ZmPIN1c*, were also showed significantly up-regulated expression after IAA treatment (Fig. 4). An ortholog of *AtABCB1* in maize, *Dwarf Brachytic2*, has been reported to be involved in auxin efflux out of meristematic regions in the shoots and roots. Meanwhile, *br2* mutant reduced auxin export out of the shoot apex [70]. The expression of *BR2*, which was renamed *ZmABCB4* in our study, was induced by IAA treatment in both the roots and shoots. These results suggested that maize auxin transporter genes may be regulated by an auxin feedback mechanism.

### *ZmLAX*, *ZmPIN*, *ZmPILS* and *ZmABCB* genes were involved in salt, drought and cold stress responses

Auxin mediates various abiotic stress responses in plants by controlling a large number of auxin-responsive genes that are thought to be involved in abiotic stress responses [78]. It has been reported that various environmental and abiotic signals can change auxin distribution by modulating trafficking and PIN protein polarities [79]. In our study, the expressions of most *ZmPIN*, *ZmPILS*, *ZmLAX* and *ZmABCB* genes were up-regulated by high salinity and drought in the shoots, but were down-regulated in the roots. The involvement of these genes in the salt and drought stress responses and their similar expression patterns suggested that the expressions of these auxin transporter encoding genes were regulated by the same physiological signal. High salinity and drought are the major causes of the changes in osmotic pressure in plant cells [80], so it is possible that these *ZmPIN*, *ZmPILS*, *ZmLAX* and *ZmABCB* genes may regulate maize responses to osmotic stress [81].

Cold stress is one of the major limiting factors on crop growth and productivity [80], and many studies have shown that there is a relationship between auxin and cold stress [82,83]. Cold stress affected plant growth and development regulation is closely linked to the intracellular auxin gradient, which is controlled by polar localization and the intracellular trafficking of auxin transporters [84]. For example, the asymmetric AtPIN3 and AtPIN2 protein redistribution and intracellular cycling were blocked by cold stress [85]. The immobilization of PINs during cold stress provides a mechanistic basis to explain the role auxin plays in regulating plant growth and development under low temperature stress [84]. The expression levels of most *ZmPIN*, *ZmPILS*, *ZmLAX* and *ZmABCB* genes were also changed by cold treatment, which suggested that these auxin transporter encoding genes may function in the mechanism that helps maize tolerate cold stress. Promoter *cis*-elements analysis showed that several stress-related motifs were present in the *ZmPIN*, *ZmPILS*, *ZmLAX* and *ZmABCB* gene promoter regions (S5 Table). It may be the genetic basis of stress expression regulation in these auxin transporter genes [86].

Some auxin transporter genes have already been reported to be involved in responses to abiotic stresses (such as high salinity, drought, cold and alkaline stress). In *Arabidopsis*, *AtPIN2* helps roots adapt to alkaline stress by modulating root tip proton secretion [18]. In salt-stressed plants, more lateral root (LR) primordia were induced during the pre-emergence to the emergence stages than the control plants. However, the stress-induced lateral root emergence and proliferation almost were abrogated in the auxin transporter mutant *aux1-7* [87]. The expression of a sorghum gene, *SbLAX4*, was dramatically reduced under various abiotic stresses [33]. These results provide genetic and physiological evidence that auxin influx carriers are involved in the response to environmental stresses. The *ZmPIN*, *ZmPILS*, *ZmLAX* and *ZmABCB* gene expression profiling changes may accelerate or decelerate the transportation of endogenous auxin. Auxin redistribution is an essential process for plant to survive in the challenge environments. For example, the *AtSOS3* gene controls lateral root developmental plasticity and low salt stress adaptation\ by regulating auxin redistribution and transport [88]. The opposite expression response patterns highlighted the dynamic auxin transport processes that occur between the shoots and roots. Auxin transport and redistribution may be required for maize is responded to abiotic stresses. Further studies, including the biological function identification and genetic analysis of each maize auxin transporter, will improve our understanding of the relationship between auxin transporters and abiotic stresses.

## Conclusions

Auxin has a fundamental role in plant development and its polar transport across cellular membranes is the key process for responses to environmental stimuli. In our study, the auxin transporter coding gene families, *ZmLAX*, *ZmPIN*, *ZmPILS* and *ZmABCB*, were well identified in maize, and the expression profiles of these genes under exogenous hormone treatments or abiotic stresses were also elucidated. The different expressions of *ZmPIN*, *ZmPILS*, *ZmLAX* and *ZmABCB* genes suggested different regulatory roles of these genes in maize tolerance to abiotic stresses. The opposite expression patterns of these auxin transporter genes in the shoots and roots under abiotic stress indicated that auxin homeostasis was an important component of the maize responses to environmental stresses.

## Supporting Information

S1 Fig The predicted transmembrane helices of the ZmLAX, ZmPIN, ZmPILS and ZmABCB Proteins. The transmembrane domains were estimated using TMHMM2 (http://www.cbs.dtu.dk/services/TMHMM/), and the red peaks show the predicted transmembrane regions of proteins.
(TIF)

S2 Fig Spatial-temporal expression patterns of auxin transporter gene families based on digital expression data. 1:Germinating Seed 24h; 2:Coleoptile 6DAS GH; 3:Coleoptile 6DAS Primary Root; 4:Stem and SAM (V1); 5:Stem and SAM (V3); 6:Stem and SAM (V4); 7:Shoot tip (V5); 8:First Internode (V5); 9:First Internode (V7); 10:Fourth Internode (V9); 11:Immature Tassel (V13); 12:Meiotic Tassel (V18); 13:Anthers (R1); 14:Whole Seedling (VE); 15:Primary Root (VE); 16:Pooled Leaves (V1); 17:Primary Root (V1); 18:Topmost Leaf (V3); 19:First Leaf (V3); 20:Tip of Stage 2 leaf (V5); 21:Base of Stage 2 leaf (V5); 22:Tip of Stage 2 leaf (V7); 23:Base of Stage 2 leaf (V7); 24:Eighth Leaf (V9); 25:Eleventh Leaf (V9); 26:Thirteenth Leaf (V9); 27:Immature Leaf (V9); 28:Thirteenth Leaf (VT); 29:Immature Cob (V18); 30:Pre-pollination Cob (R1); 31:Silks (R1); 32:Thirteenth Leaf (R2); 33:Innermost Husk (R1); 34:Innermost Husk (R2); 35:Outer Husk (R2); 36:Embryo 16DAP; 37:Embryo 18DAP; 38:Embryo 20DAP; 39:Embryo 22DAP; 40:Embryo 24DAP; 41:Endosperm 12DAP; 42:Endosperm 14DAP; 43:Endosperm 16DAP; 44:Endosperm 18DAP; 45:Endosperm 20DAP; 46:Endosperm 22DAP; 47:Endosperm 24DAP; 48:Seed 2DAP; 49:Seed 4DAP; 50:Seed 6DAP; 51:Seed 8DAP; 52:Seed 10DAP; 53:Seed 12DAP; 54:Seed 14DAP; 55:Seed 16DAP; 56:Seed 18DAP; 57:Pericarp 18DAP; 58:Seed 20DAP; 59:Seed 22DAP; 60:Seed 24DAP.
(TIF)

S1 Table *PIN*, *PILS*, *LAX* and *ABCB* family genes in *Arabidopsis* and rice. ^a^ Locus ID of gene in TAIR database (http://www.arabidopsis.org/). ^b^Accession number of corresponding protein existed in UniProt database (http://www.uniprot.org/). ^c^The locus ID of genes in TIGR Rice Genome Annotation Project Database (http://rice.plantbiology.msu.edu/). ^d^Accession number of corresponding protein sequence existed in UniProt database (http://www.uniprot.org/).
(DOCX)

S2 Table Spatial-temporal expression patterns of auxin transporter gene families based on digital expression data. (XLSX)

S3 Table The primer sequences of *ZmLAX*, *ZmPIN*, *ZmPILS* and *ZmABCB* genes. (DOCX)

S4 Table The values of the expression levels of all *ZmPIN*, *ZmPILS*, *ZmLAX* and *ZmABCB* genes. (DOCX)

S5 Table Number of stress-related *cis*-elements in the promoter regions of *ZmPIN*, *ZmPILS*, *ZmLAX* and Z*mABCB* genes. (DOCX)
