# Supplementary figures and images for: Genome-Wide Identification and Expression Profiling Analysis of ZmPIN, ZmPILS, ZmLAX and ZmABCB Auxin Transporter Gene Families in Maize (Zea mays L.) under Various Abiotic Stresses
Source: PLoS One. 2015 Mar 5;10(3):e0118751. doi: 10.1371/journal.pone.0118751 (PMC4351008; doi:10.1371/journal.pone.0118751)

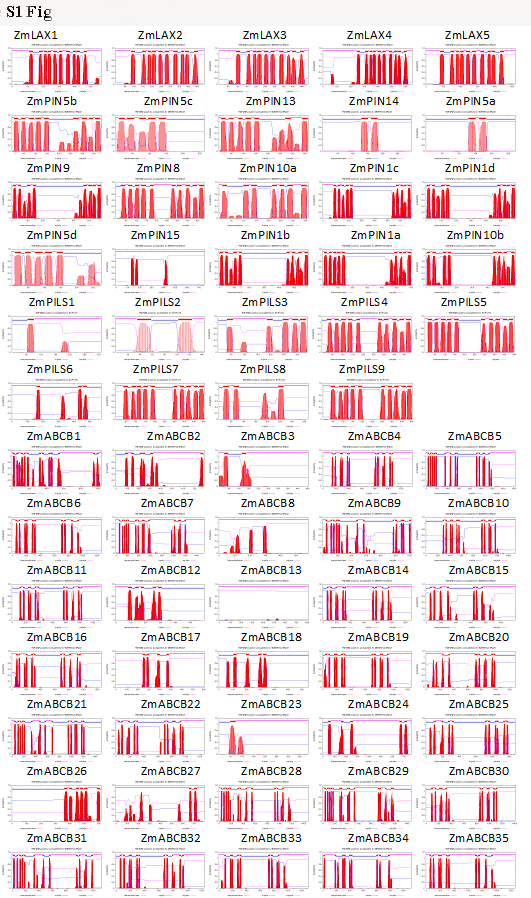

Supplement: S1 Fig — The transmembrane domains were estimated using TMHMM2 (http://www.cbs.dtu.dk/services/TMHMM/), and the red peaks show the predicted transmembrane regions of proteins. (TIF) [file pone.0118751.s001.tif]

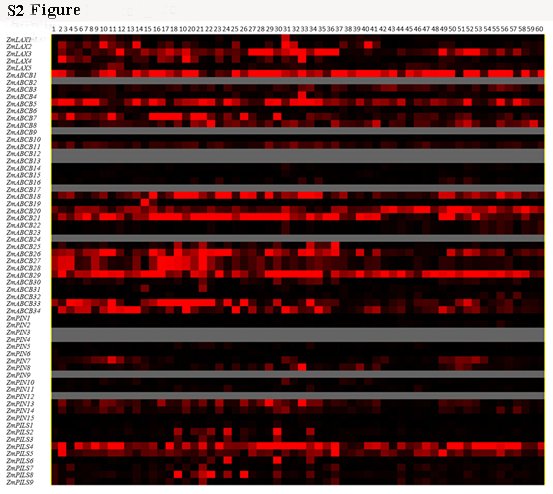

Supplement: S2 Fig — 1:Germinating Seed 24h; 2:Coleoptile 6DAS GH; 3:Coleoptile 6DAS Primary Root; 4:Stem and SAM (V1); 5:Stem and SAM (V3); 6:Stem and SAM (V4); 7:Shoot tip (V5); 8:First Internode (V5); 9:First Internode (V7); 10:Fourth Internode (V9); 11:Immature Tassel (V13); 12:Meiotic Tassel (V18); 13:Anthers (R1); 14:Whole Seedling (VE); 15:Primary Root (VE); 16:Pooled Leaves (V1); 17:Primary Root (V1); 18:Topmost Leaf (V3); 19:First Leaf (V3); 20:Tip of Stage 2 leaf (V5); 21:Base of Stage 2 leaf (V5); 22:Tip of Stage 2 leaf (V7); 23:Base of Stage 2 leaf (V7); 24:Eighth Leaf (V9); 25:Eleventh Leaf (V9); 26:Thirteenth Leaf (V9); 27:Immature Leaf (V9); 28:Thirteenth Leaf (VT); 29:Immature Cob (V18); 30:Pre-pollination Cob (R1); 31:Silks (R1); 32:Thirteenth Leaf (R2); 33:Innermost Husk (R1); 34:Innermost Husk (R2); 35:Outer Husk (R2); 36:Embryo 16DAP; 37:Embryo 18DAP; 38:Embryo 20DAP; 39:Embryo 22DAP; 40:Embryo 24DAP; 41:Endosperm 12DAP; 42:Endosperm 14DAP; 43:Endosperm 16DAP; 44:Endosperm 18DAP; 45:Endosperm 20DAP; 46:Endosperm 22DAP; 47:Endosperm 24DAP; 48:Seed 2DAP; 49:Seed 4DAP; 50:Seed 6DAP; 51:Seed 8DAP; 52:Seed 10DAP; 53:Seed 12DAP; 54:Seed 14DAP; 55:Seed 16DAP; 56:Seed 18DAP; 57:Pericarp 18DAP; 58:Seed 20DAP; 59:Seed 22DAP; 60:Seed 24DAP. (TIF) [file pone.0118751.s002.tif]
