# Supplementary material for: Genome-Wide Identification and Expression Profiling Analysis of ZmPIN, ZmPILS, ZmLAX and ZmABCB Auxin Transporter Gene Families in Maize (Zea mays L.) under Various Abiotic Stresses
Source: PLoS One. 2015 Mar 5;10(3):e0118751. doi: 10.1371/journal.pone.0118751 (PMC4351008; doi:10.1371/journal.pone.0118751)
Supplement: S1 Table — a Locus ID of gene in TAIR database (http://www.arabidopsis.org/). bAccession number of corresponding protein existed in UniProt database (http://www.uniprot.org/). cThe locus ID of genes in TIGR Rice Genome Annotation Project Database (http://rice.plantbiology.msu.edu/). dAccession number of corresponding protein sequence existed in UniProt database (http://www.uniprot.org/). (DOCX) [file pone.0118751.s003.docx]

S1 Table. *PIN*, *PILS*, *LAX* and *ABCB* family genes in *Arabidopsis* and rice

| Species | Locus in TAIR | Locus in TIGR | Accession | Gene name | Synonyms name |
| --- | --- | --- | --- | --- | --- |
| *Arabidopsis* | At1g73590 |  | Q9C6B8 | AtPIN1 |  |
|  | At5g57090 |  | Q9LU77 | AtPIN2 |  |
|  | At1g70940 |  | Q9S7Z8 | AtPIN3 |  |
|  | At2g01420 |  | Q8RWZ6 | AtPIN4 |  |
|  | At5g16530 |  | Q9FFD0 | AtPIN5 |  |
|  | At1g77110 |  | Q9SQH6 | AtPIN6 |  |
|  | At1g23080 |  | Q940Y5 | AtPIN7 |  |
|  | At5g15100 |  | Q9LFP6 | AtPIN8 |  |
|  | At1g20925 |  |  | AtPLIS1 |  |
|  | At1g71090 |  |  | AtPLIS2 |  |
|  | At1g76520 |  |  | AtPLIS3 |  |
|  | At1g76530 |  |  | AtPLIS4 |  |
|  | At2g17500 |  |  | AtPLIS5 |  |
|  | At5g01990 |  |  | AtPLIS6 |  |
|  | At5g65980 |  |  | AtPLIS7 |  |
|  | At2g38120 |  | Q96247 | AtAUX1 |  |
|  | At5g01240 |  | Q9LFB2 | AtLAX1 |  |
|  | At2g21050 |  | Q9S836 | AtLAX2 |  |
|  | At1g77690 |  | Q9CA25 | AtLAX3 |  |
|  | At2g36910 |  | Q9ZR72 | AtABCB1 | AtPGP1 |
|  | At4g25960 |  | Q8LPK2 | AtABCB2 | AtPGP2 |
|  | At4g01820 |  | Q9SYI2 | AtABCB3 | AtPGP3 |
|  | At2g47000 |  | O80725 | AtABCB4 | AtPGP4 |
|  | At4g01830 |  | Q9SYI3 | AtABCB5 | AtPGP5 |
|  | At2g39480 |  | Q8LPT1 | AtABCB6 | AtPGP6 |
|  | At5g46540 |  | Q9FHF1 | AtABCB7 | AtPGP7 |
|  | At4g18050 |  | Q9M0M2 | AtABCB9 | AtPGP9 |
|  | At1g10680 |  | Q9SGY1 | AtABCB10 | AtPGP10 |
|  | At1g02520 |  | Q9FWX7 | AtABCB11 | AtPGP11 |
|  | At1g02530 |  | Q9FWX8 | AtABCB12 | AtPGP12 |
|  | At1g27940 |  | Q9C7F8 | AtABCB13 | AtPGP13 |
|  | At1g28010 |  | Q9C7F2 | AtABCB14 | AtPGP14 |
|  | At3g28345 |  | Q9LHD1 | AtABCB15 | AtPGP15 |
|  | At3g28360 |  | Q9LSJ8 | AtABCB16 | AtPGP16 |
|  | At3g28380 |  | Q9LSJ6 | AtABCB17 | AtPGP17 |
|  | At3g28390 |  | Q9LSJ5 | AtABCB18 | AtPGP18 |
|  | At3g28860 |  | Q9LJX0 | AtABCB19 | AtPGP19 |
|  | At3g55320 |  | Q9M3B9 | AtABCB20 | AtPGP20 |
|  | At3g62150 |  | Q9M1Q9 | AtABCB21 | AtPGP21 |
|  | At3g28415 |  | Q9LSJ2 | AtABCB22 | AtPGP22 |
| rice |  | LOC_Os06g12610 | Q67UL3 | OsPIN1a |  |
|  |  | LOC_Os02g50960 | Q5SMQ9 | OsPIN1b |  |
|  |  | LOC_Os11g04190 | P0C0X5 | OsPIN1c |  |
|  |  | LOC_Os12g04000 | Q0IQA5 | OsPIN1d |  |
|  |  | LOC_Os06g44970 | Q651V6 | OsPIN2 |  |
|  |  | LOC_Os01g69070 | Q5JLM1 | OsPIN5a |  |
|  |  | LOC_Os08g41720 | Q6ZIB5 | OsPIN5b |  |
|  |  | LOC_Os09g32770 | Q0J0M0 | OsPIN5c |  |
|  |  | LOC_Os01g51780 | Q0JJV0 | OsPIN8 |  |
|  |  | LOC_Os01g58860 | Q5VQY3 | OsPIN9 |  |
|  |  | LOC_Os01g45550 | Q5VP70 | OsPIN10a |  |
|  |  | LOC_Os05g50140 | Q6L5F6 | OsPIN10b |  |
|  |  | LOC_Os01g60230 |  | OsPILS1 |  |
|  |  | LOC_Os05g40330 |  | OsPILS2 |  |
|  |  | LOC_Os08g09190 |  | OsPILS3 |  |
|  |  | LOC_Os09g31478 |  | OsPILS4 |  |
|  |  | LOC_Os09g38130 |  | OsPILS5 |  |
|  |  | LOC_Os09g38210 |  | OsPILS6 |  |
|  |  | LOC_Os01g63770 | Q5N892 | OsLAX1 |  |
|  |  | LOC_Os03g14080 | Q10P71 | OsLAX2 |  |
|  |  | LOC_Os05g37470 | Q688J2 | OsLAX3 |  |
|  |  | LOC_Os10g05690 | Q7XGU4 | OsLAX4 |  |
|  |  | LOC_Os11g06820 | Q53JG7 | OsLAX5 |  |
|  |  | LOC_Os01g18670 | Q0JNH5 | OsABCB1 | OsPGP1 |
|  |  | LOC_Os01g34970 | Q0JM60 | OsABCB2 | OsPGP2 |
|  |  | LOC_Os01g35030 | C7IX17 | OsABCB3 | OsPGP3 |
|  |  | LOC_Os01g50080 | Q0JK52 | OsABCB4 | OsPGP4 |
|  |  | LOC_Os01g50100 | Q0JK51 | OsABCB5 | OsPGP5 |
|  |  | LOC_Os01g50160 | Q5N8L3 | OsABCB6 | OsPGP6 |
|  |  | LOC_Os01g52550 | Q8GU81 | OsABCB7 | OsPGP7 |
|  |  | LOC_Os01g74470 | Q0JFK0 | OsABCB8 | OsPGP8 |
|  |  | LOC_Os02g09720 | Q6YUU5 | OsABCB9 | OsPGP9 |
|  |  | LOC_Os02g21750 | C7IY55 | OsABCB10 | OsPGP10 |
|  |  | LOC_Os02g46680 | Q8GU75 | OsABCB11 | OsPGP11 |
|  |  | LOC_Os03g08380 | Q8H7L0 | OsABCB12 | OsPGP12 |
|  |  | LOC_Os03g17180 | Q0DSY8 | OsABCB13 | OsPGP13 |
|  |  | LOC_Os04g38570 | Q0JCP1 | OsABCB14 | OsPGP14 |
|  |  | LOC_Os04g40570 | Q0JCB0 | OsABCB15 | OsPGP15 |
|  |  | LOC_Os04g54930 | Q0J9M8 | OsABCB16 | OsPGP16 |
|  |  | LOC_Os05g04610 | Q5KQM4 | OsABCB17 | OsPGP17 |
|  |  | LOC_Os05g47490 | Q0DG83 | OsABCB18 | OsPGP18 |
|  |  | LOC_Os05g47500 | B9FHL5 | OsABCB19 | OsPGP19 |
|  |  | LOC_Os08g05690 | Q8GU79 | OsABCB20 | OsPGP20 |
|  |  | LOC_Os08g05710 | Q8GU80 | OsABCB21 | OsPGP21 |
|  |  | LOC_Os08g45030 | Q7EZL3 | OsABCB22 | OsPGP22 |
