# Supplementary material for: Genome-Wide Identification and Expression Profiling Analysis of ZmPIN, ZmPILS, ZmLAX and ZmABCB Auxin Transporter Gene Families in Maize (Zea mays L.) under Various Abiotic Stresses
Source: PLoS One. 2015 Mar 5;10(3):e0118751. doi: 10.1371/journal.pone.0118751 (PMC4351008; doi:10.1371/journal.pone.0118751)
Supplement: S3 Table — (DOCX) [file pone.0118751.s005.docx]

S3 Table. The primer sequences of *ZmLAX*, *ZmPIN*, *ZmPILS* and *ZmABCB* genes

| ZmLAX1-dn:CCCAGTTGCGAGAACGAGTAG |
| --- |
| ZmLAX2-up:GGGCGACCAACATACTCTACAC |
| ZmLAX2-dn:TGGATGAGCATGAGCACCAC |
| ZmLAX3-up:TCACCGTCTACATCATCCCG |
| ZmLAX3-dn:GAACGTGTCGATCTGCCTCA |
| ZmLAX4-up: TTGAGGTTCTGGATGGGCTAC |
| ZmLAX4-dn: TCCACGTCCGCTTGTCATAC |
| ZmLAX5-up:ATTGAACGCAGCGGAGAAG |
| ZmLAX5-dn:TCGGGCACTGGTAGCACTT |
| ZmPIN5b-up:ACGCTGGTGGCGAACTTCT |
| ZmPIN5b-dn:TTGCCGTACATGGCGAAGA |
| ZmPIN5c-up:TACTTCGCCGTGCCCTTCT |
| ZmPIN5c-dn:ACGACGAGCGTGTTGTTGAG |
| ZmPIN13-up:TGTCCACCAACGACCCGTA |
| ZmPIN13-dn:CGACGCCACCGAGAAGTT |
| ZmPIN14-up:ACCAACGACCCGTACCACAT |
| ZmPIN14-dn:ACCCACTTGATCGGCGACT |
| ZmPIN5a-up:TGACGCTCCTCCTCTTCGT |
| ZmPIN5a-dn:TGCGCCACCACCTTGAT |
| ZmPIN9-up:CCAGATTCCGAACACCTATGC |
| ZmPIN9-dn:CCTATCAGGAACTTGAGGAGCA |
| ZmPIN8-up:TGTGCTGCGAGGAGAAGTT |
| ZmPIN8-dn:CAGCCCGAAACTCAAACAG |
| ZmPIN10a-up: GAACGAGAACAACGGTGCAG |
| ZmPIN10a-dn: TGGAGATGTGCCACCGGAAC |
| ZmPIN1c-up:TCGTGCTCCAGTGCATCATC |
| ZmPIN1c-dn:ACGTGTATCCTGCCGTCCTC |
| ZmPIN1d-up:ACAGCAGCAGGACGAAGAAC |
| ZmPIN1d-dn:CGATGAGGCTGGAGTAGGTG |
| ZmPIN5d-up:TCGCTGTCCACGCTAACCA |
| ZmPIN5d-dn:ATGGCGGCCTTTCTGGTC |
| ZmPIN15-up:AAGAGCGGTTTCGTTTCG |
| ZmPIN15-dn:ATTCACCCAGGCAGCAAG |
| ZmPIN1b-up:TCATCCGCAACCCAAACAC |
| ZmPIN1b-dn:ATGAACAGCCCGAGACTGAAC |
| ZmPIN1a-up:GCTGCTGTCCTTCCACTTC |
| ZmPIN1a-dn:GCCGTACATGCCCTTGA |
| ZmPIN10b-up:GGCATCAACCGCTTCGTC |
| ZmPIN10b-dn:TGTTGGGCAGGGTGGACA |
| ZmPILS1-up:CATGAGCACGCGAAAGAAGA |
| ZmPILS1-dn:TCGCCAACGATTAGGGATTT |
| ZmPILS2-up:ATAATTGGGTTCGTTGTTGG |
| ZmPILS2-dn:ACATAGCAGTACAGCGGGTC |
| ZmPILS3-up:CGGAGATGACCGTCTATGAG |
| ZmPILS3-dn:GCTCTGTAGCCGACGATTAG |
| ZmPILS4-up:CCACCAGGACAGACCTTTGA |
| ZmPILS4-dn:AATGGGATAACACCGATTGC |
| ZmPILS5-up:GCCTCATCTTCGTCCACCTC |
| ZmPILS5-dn:AAGAATGACCGCAACCCACT |
| ZmPILS6-up:AGCATGAGCAAGCGAAAGAA |
| ZmPILS6-dn:CGATGAACTGGTGGATAGCG |
| ZmPILS7-up:ACATCAAAGGTGGAGGAAGG |
| ZmPILS7-dn:ATGGTTGAAGGAGCGAAGAG |
| ZmPILS8-up:TCGTGGATTTGCATTCACCT |
| ZmPILS8-dn:AAGACTGCCCTTGGAGCATC |
| ZmPILS9-up:GCTCAGGAAGCGGTAGAGGA |
| ZmPILS9-dn:ATCAACGTGACGGACGGAAT |
| ZmABCB1-up:GACTGTCAAACGCCTCACG |
| ZmABCB1-dn:TTTGCCACAGGAACAGAGG |
| ZmABCB2-up:TGTTGGTAGAAATGGCTCCG |
| ZmABCB2-dn:TTTCCCGAATGCTCAAACTC |
| ZmABCB3-up:CAAGTATGACACCGTAGTAGGAGA |
| ZmABCB3-dn:CGATCGGAAGATAAGGATTTC |
| ZmABCB4-up:ATGACAGCAAGAAGCCCACC |
| ZmABCB4-dn:CGACGACGAGGAAGTAGAAGG |
| ZmABCB5-up:CTCAGTGGTCATTCGTGGAT |
| ZmABCB5-dn:CTGGGAGTAGGTCAAGGTCA |
| ZmABCB6-up:GACTTCTTTCGTCGTTGGAT |
| ZmABCB6-dn:GATCTTGCTTTGAGCGTTG |
| ZmABCB7-up:AAGGGCTCCAATCCTACACC |
| ZmABCB7-dn:CCTGAAAGAAAGCCACTCCA |
| ZmABCB8-up:CCGTGCTCAACGTCATTCC |
| ZmABCB8-dn:CCCATTGTTTGCCTTCACAG |
| ZmABCB9-up:CAGTGGTTGGGATGGTAGGAT |
| ZmABCB9-dn:CCAGGCAGCAAGAATGAATG |
| ZmABCB10-up:TGTCAGGAGGGCAGAAGCA |
| ZmABCB10-dn:ACAGACGGTGAGCCACTACGA |
| ZmABCB11-up:TGGGAGTGGCAAATCAAC |
| ZmABCB11-dn:TGCCCTTATCGTGTCGTT |
| ZmABCB12-up: CTTGTACATTAGGACCTTGT |
| ZmABCB12-dn: TGTTGCTGCTTGATTAAGTC |
| ZmABCB13-up:CAGAAGCGGTTGTGGTAAATC |
| ZmABCB13-dn:AAGCAATGTTGTCCCTGACAC |
| ZmABCB14-up:GCAATCGTAAAGAACCCTAGG |
| ZmABCB14-dn:TCAACCACCTTCCCGTGT |
| ZmABCB15-up:AAGCCAGAGGCAGATGACA |
| ZmABCB15-dn:GAAGGCAACAATGATAGCAAC |
| ZmABCB16-up:AAGAAGAAGAAGGCGGAAGAG |
| ZmABCB16-dn:CCGAAGAAGATGAAGAAGACG |
| ZmABCB17-up:TTTATGCTCGCCGTGTCT |
| ZmABCB17-dn:TCGGATGCTTTCCTTGC |
| ZmABCB18-up:GCTTCTGTCGGGTCATCAA |
| ZmABCB18-dn:GCTTGCGCCTTATCTTCTG |
| ZmABCB19-up:GCTAAGGACGCCAACATCG |
| ZmABCB19-dn:CTTGCTGGTTTCGGACTGC |
| ZmABCB20-up:TTATCTGAAGCAACAGGTCGTAG |
| ZmABCB20-dn:TGTGGCAGAAATCGAGGC |
| ZmABCB21-up:AAGGGACCATTAGGAGCAACA |
| ZmABCB21-dn:TGGGAATCAACGGAAGCAG |
| ZmABCB22-up:TCGGATGAAGGTGAAGAAAG |
| ZmABCB22-dn:CAATGTCCAGAACCGTGAAT |
| ZmABCB23-up:GACTCAGCCAAGGCAAATG |
| ZmABCB23-dn:GGACGGGATGGGTATTTG |
| zmABCB24-up:CCTCCATTTGTTGTTGCTGC |
| zmABCB24-dn:ACGATCCCTTCCTTGACTGC |
| ZmABCB25-up:TGTTGAGCGGTTCTACGATC |
| ZmABCB25-dn:CCCTCCTGACCTCTTCTACTG |
| ZmABCB26-up:TACAAGAGGGCGTGGAAGG |
| ZmABCB26-dn:TCAGCGTGAAGGCGAACA |
| ZmABCB27-up:GTTAAATGCCTCTGCTGACC |
| ZmABCB27-dn:CAGAACCAACCTCGCCTAC |
| ZmABCB28-up:CGTTCGTCGCTTGTTCCTC |
| ZmABCB28-dn:GTTCCAGTTCCGCCATCAC |
| ZmABCB29-up:CCAGATCGCATCCACATATTG |
| ZmABCB29-dn:TTGAATACGGAGTCCATTAGCC |
| ZmABCB30-up:AAACCACCCAATGTCTACGG |
| ZmABCB30-dn:AGATCTCGACCATCCAGCAG |
| ZmABCB31-up:GCGGTACTACATTGACACGG |
| ZmABCB31-dn:GCGAAAGCAAAGCCACTC |
| ZmABCB32-up:ACCGTCTTCTCCTTCGTTGG |
| ZmABCB32-dn:CCACCTCCTGTTGCCTTGTT |
| ZmABCB33-up:TCACTCCCACGCACCTGTT |
| ZmABCB33-dn:GCTGCTGCTGTTGCTTCCT |
| ZmABCB34-up:ATTGCTGGACTTGCGGTTAC |
| ZmABCB34-dn:GTGGGCGGTAGTGGTTAACTA |
| ZmABCB35-up:GCCAGAAACCCTCCATAGTCA |
| ZmABCB35-dn:TGCTCTTTCCAGACCCACTG |
