# Supplementary material for: Genome-Wide Identification and Expression Profiling Analysis of ZmPIN, ZmPILS, ZmLAX and ZmABCB Auxin Transporter Gene Families in Maize (Zea mays L.) under Various Abiotic Stresses
Source: PLoS One. 2015 Mar 5;10(3):e0118751. doi: 10.1371/journal.pone.0118751 (PMC4351008; doi:10.1371/journal.pone.0118751)
Supplement: S4 Table — (DOCX) [file pone.0118751.s006.docx]

S4 Table. The values of the expression levels of all *ZmPIN*, *ZmPILS*, *ZmLAX* and *ZmABCB* genes

|  | L | S | R | F |
| --- | --- | --- | --- | --- |
| ZmPIN5b | 0.206003 | 1.364131 | -0.03395 | 0.8383 |
| ZmPIN5c | 1.984419 | 2.221288 | 0.196001 | -0.6099 |
| ZmPIN13 | 0.92195 | 1.890604 | 1.187586 | 2.339807 |
| ZmPIN14 | 1.060667 | 1.471482 | 1.151369 | 1.162834 |
| ZmPIN5a | 1.866385 | 1.600754 | 0.09444 | 2.333506 |
| ZmPIN9 | 0.731974 | 1.457443 | 1.797724 | -0.94441 |
| ZmPIN8 | 0.369929 | 2.376936 | 0.978303 | 0.828992 |
| ZmPIN10a | 0.894766 | 2.005456 | -0.21011 | -0.93802 |
| ZmPIN1c | 1.0464 | 3.036985 | 1.407579 | -0.34232 |
| ZmPIN1d | 1.254429 | 0.315664 | -0.40502 | 1.163376 |
| ZmPIN5d | 1.0561 | 1.254963 | 0.568072 | -1.87456 |
| ZmPIN15 | 1.688343 | -0.37107 | 1.309314 | -1.57353 |
| ZmPIN1b | 2.003346 | 4.052859 | 2.017065 | 1.463864 |
| ZmPIN1a | 1.818724 | 3.384303 | 1.465612 | -0.94437 |
| ZmPIN10b | 2.004568 | 2.791675 | 1.166654 | 1.436771 |
| ZmPILS1 | 3.004568 | 3.791675 | 2.166654 | 2.436771 |
| ZmPILS2 | 0.255008 | 1.949211 | 1.238061 | 2.737804 |
| ZmPILS3 | 2.999956 | 3.15896 | 2.126214 | -0.61002 |
| ZmPILS4 | -1.89763 | 1.628652 | -2.44494 | 0.664353 |
| ZmPILS5 | 4.026412 | 5.089064 | 4.465486 | 3.64147 |
| ZmPILS6 | 4.491851 | 5.364077 | 4.479881 | 3.066503 |
| ZmPILS7 | 2.793383 | 3.320132 | 2.405309 | 1.829263 |
| ZmPILS8 | 2.498499 | 3.634261 | 4.351761 | 0.69171 |
| ZmPILS9 | -0.79789 | -0.2503 | -0.98006 | 0.968658 |
| ZmLAX1 | 0.651466 | 2.635717 | 0.719025 | 0.560719 |
| ZmLAX2 | 0.619213 | 3.25184 | 1.438658 | -1.57357 |
| ZmLAX3 | 2.710321 | 4.506623 | 2.857748 | -2.20904 |
| ZmLAX4 | 0.300307 | 3.193004 | 1.291862 | -1.24543 |
| ZmLAX5 | 2.218884 | 3.320573 | 2.303309 | -2.1485 |
| ZmABCB1 | 1.800607 | 2.751974 | 2.161993 | -1.24541 |
| ZmABCB2 | 2.019293 | 2.522377 | 1.87547 | 1.129386 |
| ZmABCB3 | 1.720215 | 1.950776 | 1.448903 | 0.52821 |
| ZmABCB4 | 1.313997 | 1.230422 | -0.29855 | -0.33602 |
| ZmABCB5 | 2.734776 | 2.874165 | 2.527445 | -0.06843 |
| ZmABCB6 | 1.245077 | 2.61413 | 1.982307 | 1.129386 |
| ZmABCB7 | 3.781078 | 2.686608 | 3.299716 | 1.464747 |
| ZmABCB8 | 1.54888 | 1.283548 | 0.385302 | -0.31163 |
| ZmABCB9 | 1.75544 | 1.794674 | 1.149693 | 2.975309 |
| ZmABCB10 | 1.181152 | 1.32015 | -0.27992 | 1.494874 |
| ZmABCB11 | 2.965459 | 3.278144 | 3.267585 | -0.00154 |
| ZmABCB12 | 0.943986 | 1.823409 | 0.677537 | 0.835623 |
| ZmABCB13 | 1.46178 | 2.51935 | 2.786973 | 0.533681 |
| ZmABCB14 | 0.705959 | 1.151148 | 1.128855 | -0.04129 |
| ZmABCB15 | 0.306369 | 0.858827 | 0.11884 | -0.67044 |
| ZmABCB16 | 1.446362 | 1.198553 | 0.04198 | -0.91093 |
| ZmABCB17 | 2.063335 | 1.940013 | 1.78764 | 1.196282 |
| ZmABCB18 | 2.993968 | 1.452439 | 1.833235 | -1.87458 |
| ZmABCB19 | 0.609999 | -0.19587 | -0.49809 | -1.24541 |
| ZmABCB20 | 1.462531 | 1.970886 | 1.716706 | 1.470219 |
| ZmABCB21 | 3.326776 | 2.976177 | 3.190025 | 2.941921 |
| ZmABCB22 | -0.77662 | -0.1782 | -1.45848 | -0.9709 |
| ZmABCB23 | 0.746735 | 1.563401 | 0.725884 | -0.06804 |
| ZmABCB24 | 2.210641 | 2.883489 | 1.327289 | 1.136289 |
| ZmABCB25 | 0.79348 | 0.979522 | 0.811816 | -0.30887 |
| ZmABCB26 | 2.30226 | 2.018343 | 1.443895 | -0.00784 |
| ZmABCB27 | 1.772817 | 1.81243 | 1.597255 | 0.868134 |
| ZmABCB28 | 2.070004 | 1.799469 | 1.133076 | 0.528208 |
| ZmABCB29 | 3.41657 | 2.710093 | 2.888876 | -0.91093 |
| ZmABCB30 | 3.129763 | 3.40399 | 2.858462 | -1.54644 |
| ZmABCB31 | 2.389954 | 2.246573 | 1.692907 | -0.03493 |
| ZmABCB32 | 1.950131 | 1.688067 | 1.269866 | -0.94437 |
| ZmABCB33 | 1.339673 | 0.813797 | 0.910811 | -0.67679 |
| ZmABCB34 | -3.07913 | -1.90482 | -2.2935 | -1.54646 |
| ZmABCB35 | 1.229063 | 2.180677 | -1.93464 | -0.33596 |
| ZmACTIN | 3 | 3.0086 | 3.003461 | 2.997386 |
