# Supplementary material for: Genome-Wide Identification and Expression Profiling Analysis of ZmPIN, ZmPILS, ZmLAX and ZmABCB Auxin Transporter Gene Families in Maize (Zea mays L.) under Various Abiotic Stresses
Source: PLoS One. 2015 Mar 5;10(3):e0118751. doi: 10.1371/journal.pone.0118751 (PMC4351008; doi:10.1371/journal.pone.0118751)
Supplement: S5 Table — (DOCX) [file pone.0118751.s007.docx]

S5 Table. Number of stress-related *cis*-elements in the promoter regions of *ZmPIN*, *ZmPILS*, *ZmLAX* and Z*mABCB* genes

|  |  | DRE/CRT | ABRE | AuxRE | SARE | G-box | W-box | CG-box | P1BS | SURE |
| --- | --- | --- | --- | --- | --- | --- | --- | --- | --- | --- |
| *ZmLAX1* | GRMZM2G149481 | 0 | 0 | 0 | 0 | 0 | 2 | 0 | 0 | 0 |
| *ZmLAX2* | GRMZM2G129413 | 0 | 0 | 1 | 1 | 1 | 1 | 1 | 0 | 3 |
| *ZmLAX3* | GRMZM2G127949 | 0 | 0 | 0 | 1 | 0 | 1 | 0 | 0 | 1 |
| *ZmLAX4* | GRMZM2G045057 | 0 | 1 | 0 | 2 | 0 | 0 | 1 | 1 | 0 |
| *ZmLAX5* | GRMZM2G067022 | 0 | 0 | 0 | 2 | 0 | 1 | 1 | 0 | 0 |
| *ZmABCB1* | GRMZM5G820122 | 1 | 0 | 1 | 0 | 0 | 1 | 9 | 2 | 2 |
| *ZmABCB2* | GRMZM2G401769 | 0 | 1 | 0 | 0 | 0 | 3 | 4 | 0 | 5 |
| *ZmABCB3* | GRMZM2G032936 | 0 | 0 | 1 | 0 | 2 | 0 | 3 | 0 | 4 |
| *ZmABCB4* | GRMZM2G315375 | 0 | 0 | 0 | 2 | 0 | 0 | 5 | 1 | 2 |
| *ZmABCB5* | GRMZM2G084181 | 1 | 0 | 0 | 1 | 0 | 0 | 6 | 0 | 0 |
| *ZmABCB6* | GRMZM2G072850 | 0 | 0 | 0 | 1 | 1 | 1 | 1 | 0 | 3 |
| *ZmABCB7* | GRMZM2G032218 | 0 | 1 | 0 | 3 | 1 | 0 | 7 | 0 | 1 |
| *ZmABCB8* | GRMZM2G388539 | 0 | 0 | 0 | 0 | 0 | 0 | 9 | 0 | 1 |
| *ZmABCB9* | GRMZM2G365957 | 0 | 1 | 0 | 2 | 0 | 4 | 5 | 0 | 0 |
| *ZmABCB10* | GRMZM2G167658 | 0 | 2 | 1 | 2 | 0 | 1 | 3 | 1 | 3 |
| *ZmABCB11* | GRMZM2G119894 | 2 | 0 | 0 | 1 | 1 | 3 | 9 | 1 | 4 |
| *ZmABCB12* | GRMZM2G049351 | 0 | 1 | 0 | 0 | 1 | 0 | 4 | 1 | 1 |
| *ZmABCB13* | GRMZM2G025860 | 0 | 0 | 1 | 1 | 0 | 1 | 1 | 1 | 0 |
| *ZmABCB14* | GRMZM2G086730 | 1 | 0 | 0 | 0 | 0 | 0 | 2 | 0 | 0 |
| *ZmABCB15* | GRMZM2G441722 | 0 | 0 | 0 | 2 | 0 | 0 | 0 | 0 | 3 |
| *ZmABCB16* | GRMZM2G004748 | 0 | 0 | 0 | 0 | 0 | 3 | 1 | 0 | 2 |
| *ZmABCB17* | GRMZM2G146034 | 1 | 0 | 0 | 3 | 0 | 0 | 12 | 0 | 0 |
| *ZmABCB18* | GRMZM2G072071 | 0 | 0 | 0 | 0 | 1 | 0 | 2 | 0 | 1 |
| *ZmABCB19* | GRMZM5G843192 | 1 | 0 | 1 | 1 | 0 | 0 | 2 | 0 | 1 |
| *ZmABCB20* | GRMZM5G832772 | 1 | 0 | 2 | 0 | 1 | 0 | 0 | 0 | 1 |
| *ZmABCB21* | GRMZM2G142870 | 0 | 1 | 0 | 0 | 0 | 1 | 4 | 1 | 1 |
| *ZmABCB22* | GRMZM2G082385 | 0 | 0 | 0 | 1 | 0 | 2 | 4 | 0 | 2 |
| *ZmABCB23* | GRMZM2G153961 | 0 | 0 | 0 | 0 | 0 | 0 | 3 | 0 | 1 |
| *ZmABCB24* | GRMZM5G843537 | 1 | 0 | 0 | 0 | 0 | 1 | 0 | 0 | 2 |
| *ZmABCB25* | GRMZM2G014089 | 0 | 0 | 0 | 1 | 0 | 0 | 2 | 0 | 2 |
| *ZmABCB26* | GRMZM5G874756 | 0 | 0 | 0 | 0 | 0 | 1 | 1 | 0 | 0 |
| *ZmABCB27* | GRMZM2G081573 | 3 | 1 | 0 | 2 | 1 | 0 | 6 | 1 | 1 |
| *ZmABCB28* | GRMZM2G111903 | 0 | 1 | 0 | 3 | 4 | 1 | 14 | 0 | 2 |
| *ZmABCB29* | GRMZM2G113203 | 0 | 0 | 1 | 1 | 0 | 1 | 4 | 1 | 2 |
| *ZmABCB30* | GRMZM5G891159 | 0 | 0 | 1 | 1 | 0 | 0 | 1 | 0 | 2 |
| *ZmABCB31* | GRMZM2G361256 | 2 | 0 | 0 | 3 | 0 | 1 | 7 | 1 | 2 |
| *ZmABCB32* | GRMZM2G333183 | 0 | 0 | 0 | 0 | 0 | 0 | 5 | 3 | 1 |
| *ZmABCB33* | GRMZM2G111462 | 1 | 0 | 0 | 2 | 0 | 0 | 5 | 3 | 3 |
| *ZmABCB34* | GRMZM2G413774 | 0 | 1 | 1 | 3 | 1 | 0 | 3 | 0 | 0 |
| *ZmABCB35* | GRMZM2G085236 | 0 | 0 | 1 | 0 | 0 | 0 | 3 | 1 | 0 |
| *ZmPIN5b* | GRMZM2G148648 | 0 | 0 | 0 | 1 | 0 | 0 | 0 | 0 | 0 |
| *ZmPIN5c* | GRMZM2G040911 | 1 | 0 | 0 | 0 | 0 | 1 | 10 | 0 | 2 |
| *ZmPIN13* | GRMZM2G064941 | 0 | 0 | 1 | 0 | 1 | 1 | 4 | 0 | 0 |
| *ZmPIN14* | GRMZM2G471745 | 0 | 0 | 1 | 2 | 1 | 4 | 8 | 0 | 0 |
| *ZmPIN5a* | GRMZM2G025742 | 4 | 0 | 0 | 1 | 0 | 0 | 0 | 0 | 0 |
| *ZmPIN9* | GRMZM5G859099 | 2 | 0 | 0 | 1 | 0 | 0 | 5 | 1 | 0 |
| *ZmPIN8* | GRMZM5G839411 | 1 | 0 | 1 | 1 | 0 | 0 | 2 | 0 | 2 |
| *ZmPIN10a* | GRMZM2G126260 | 0 | 0 | 0 | 1 | 0 | 3 | 2 | 0 | 1 |
| *ZmPIN1c* | GRMZM2G149184 | 1 | 0 | 0 | 0 | 0 | 0 | 1 | 2 | 2 |
| *ZmPIN1d* | GRMZM2G171702 | 2 | 0 | 2 | 1 | 0 | 0 | 5 | 0 | 2 |
| *ZmPIN5d* | GRMZM2G175983 | 4 | 0 | 0 | 1 | 0 | 0 | 0 | 0 | 0 |
| *ZmPIN15* | GRMZM2G021364 | 1 | 1 | 1 | 2 | 2 | 2 | 2 | 0 | 0 |
| *ZmPIN1b* | GRMZM2G074267 | 2 | 0 | 0 | 1 | 0 | 1 | 1 | 1 | 0 |
| *ZmPIN1a* | GRMZM2G098643 | 0 | 0 | 0 | 0 | 0 | 3 | 1 | 0 | 0 |
| *ZmPIN10b* | GRMZM2G160496 | 0 | 0 | 1 | 1 | 0 | 0 | 7 | 0 | 1 |
| *ZmPILS1* | GRMZM2G070563 | 0 | 0 | 1 | 0 | 0 | 5 | 0 | 0 | 1 |
| *ZmPILS2* | GRMZM2G331322 | 1 | 0 | 0 | 1 | 0 | 0 | 2 | 0 | 1 |
| *ZmPILS3* | GRMZM2G112598 | 0 | 0 | 1 | 1 | 0 | 0 | 4 | 1 | 1 |
| *ZmPILS4* | GRMZM2G050088 | 4 | 0 | 0 | 1 | 0 | 0 | 0 | 0 | 0 |
| *ZmPILS5* | GRMZM2G030125 | 0 | 0 | 0 | 1 | 1 | 0 | 2 | 1 | 3 |
| *ZmPILS6* | GRMZM2G475148 | 0 | 0 | 1 | 1 | 0 | 1 | 9 | 4 | 4 |
| *ZmPILS7* | GRMZM2G043254 | 0 | 0 | 1 | 1 | 0 | 0 | 0 | 0 | 0 |
| *ZmPILS8* | GRMZM2G072632 | 0 | 0 | 0 | 2 | 0 | 1 | 1 | 0 | 2 |
| *ZmPILS9* | GRMZM2G007481 | 0 | 0 | 1 | 1 | 0 | 0 | 0 | 0 | 0 |
